# Instruments for screening and assessing oral language in preschool children via digital health: a scoping review

**DOI:** 10.1590/2317-1782/e20250007en

**Published:** 2026-03-02

**Authors:** Rhadimylla Nágila Pereira, Karinna Veríssimo Meira Taveira, Joseli Soares Brazorotto

**Affiliations:** 1 Programa Associado de Pós-graduação em Fonoaudiologia, Universidade Federal do Rio Grande do Norte – UFRN - Natal (RN), Brasil.; 2 Departamento de Morfologia, Universidade Federal do Rio Grande do Norte – UFRN - Natal (RN), Brasil.; 3 Departamento de Fonoaudiologia, Universidade Federal do Rio Grande do Norte – UFRN - Natal (RN), Brasil.

**Keywords:** Child Language, Language Development Disorders, Screening, Language Tests, Telehealth

## Abstract

**Purpose:**

the unequal distribution of specialized professionals has motivated research in digital health to address the needs of oral language care in early childhood, considering that language delays are frequent concerns at this stage. This scoping review investigated: “Which instruments are applicable for oral language screening and assessment in early childhood using digital health?”.

**Research strategies:**

the databases consulted were PubMed, Web of Science, LILACS, Embase, and Scopus, in addition to grey literature through Google Scholar and ProQuest Dissertations and Theses.

**Selection criteria:**

Studies were included if they used oral language screening and assessment tests delivered via teleconsultation, mobile applications, or web-based tools, with no restrictions on time, language, or ethnicity, in clinical, educational, or non-clinical contexts.

**Data analysis:**

Of the 2,435 studies identified, 20 met the inclusion criteria.

**Results:**

Eleven studies investigated the screening of language skills and nine addressed language assessment through digital means. Fourteen studies adapted previously validated face-to-face tests for online use; four developed specific tools for teleconsultation; and two conducted synchronous or asynchronous observations without standardized protocols. Most studies were carried out in clinical contexts with speech-language pathologists, although community health workers also participated in some cases. Videoconferencing was the most frequently used digital modality.

**Conclusion:**

The findings of this review reinforce the feasibility of telehealth for screening and assessing oral language in early childhood, highlighting the need for further research to consolidate evidence-based practices in speech-language telehealth.

## INTRODUCTION

The use of oral language screening and assessment tools in healthcare services has the potential to improve referrals to specialized care consequently, reducing waiting lists, and also minimizing parental anxiety^(1,2)^.

This is particularly relevant when considering the challenges imposed by the reduced number of healthcare professionals and specialized centers, as well as their unequal distribution^(3-8)^. The validation of oral language screening and assessment instruments is essential for accurate speech-language diagnoses, fostering expanded access to services^(9-22)^.

In this scenario, the use of digital health, particularly teleconsultations, has gained importance, as it enables users to overcome geographical barriers flexibly and conveniently, while contributing to continuity of care and resource savings^(23-28)^.

In Speech-Language Pathology, studies across different specialties suggest that remote care contributes to ensuring continuity of care, patient autonomy, and resource savings, while also indicating similar efficiency compared to in-person care^(29-32)^.

Although challenges such as lack of infrastructure and digital resources, limited professional skills, and difficulties in child care delivery have been reported^(33-35)^, the use of telehealth in child speech and language has been studied in the contexts of screening, assessment, and intervention, with favorable and promising results^(36-41)^. However, it is still necessary to map the screening and assessment protocols that can be applied via telehealth.

Given this scenario and the potential benefits of digital tools in screening and assessing oral language in children, this scoping review aims to map oral language screening and assessment instruments for children aged 0 to 6 years, using digital health resources. Thus, it provides a comprehensive overview of instruments applicable for screening and assessing oral language in this age group.

## METHODS

### Protocol

This research comprises a scoping review, conducted according to the methodological framework developed by the Joanna Briggs Institute (JBI)^(42)^ and the recommendations of the Preferred Reporting Items for Systematic Reviews and Meta-Analyses extension for Scoping Reviews (PRISMA-ScR) checklist^(43)^.

The review followed the six stages proposed by the JBI protocol: formulation of the research question; identification of relevant studies; study selection; data extraction; data mapping; grouping, summarizing, and reporting/describing the findings.

This study sought to answer the following question: What are the instruments applicable for oral language screening and assessment in early childhood via digital health?

For the formulation of this question, the “PCC” acronym was used:

- Population: children aged 0 to 6 years
- Concept: application of language screening and assessment tests and protocols through digital health tools
- Context: clinical, educational, and non-clinical settings

The protocol was registered on the platform https://osf.io/, under the DOI identifier 10.17605/OSF.IO/3VWJU.

### Eligibility criteria

The inclusion criteria comprised studies that employed oral language screening and assessment tests in early childhood via digital health, namely teleconsultation, applications, and web-based tools, without restrictions on time, language, or ethnicity.

The following types of studies were excluded: studies focused on the assessment of bilingual children; systematic reviews, meta-analyses; correspondences, book publications, guidelines, websites, blogs, as well as titles and abstracts not aligned with the scope of this review.

### Information sources and search strategy

Searches were conducted in the databases PubMed, Web of Science, LILACS, Embase, Scopus, and in gray literature: Google Scholar and ProQuest Dissertations and Theses.

The search strategy combined the following keywords and Boolean operators: “Language Development Disorders” OR “language delay” AND “screening” OR “language tests” OR “standardized assessment” AND “telehealth” OR “teleconsultation” AND “child preschool” OR “toddler.” The complete strategies for each database, as well as for gray literature, are detailed in Appendix 1 (Appendix 1).

### Study selection

Phase 1: The search was conducted in a single day, and after duplicate removal in Mendeley, the files from each database were uploaded into Rayyan (https://www.rayyan.ai/)^(44)^ for initial screening.

The screening of articles was performed by two reviewers, both with experience in child oral language, following two selection phases to verify eligibility. Before article selection, the first 100 records from the PubMed search were uploaded into Rayyan for reviewer calibration, achieving a kappa of 1.0, indicating perfect agreement in the initial analysis.

Phase 1 consisted of title and abstract screening, and in cases of disagreement, a consensus meeting was held to decide on the inclusion of conflicting articles in the next phase.

Phase 2: At this stage, all selected articles were read in full, and data were extracted using a predesigned form.

Data extraction was organized into a summary table (Table 1) and in Charts 1 and 2. Table 1 presents instrument data: name, language skills assessed, whether the protocol corresponds to screening or assessment, whether it was adapted or specific, and study reference.

**Table 1:** Instruments for screening and assessment of oral language in early childhood via digital health

Instrument	Skills Assessed	Screening or Assessment	Application (professional/application time)	Adapted or Specific	Authors
Parents’ Evaluation of Developmental Status (PEDS)	Receptive and Expressive Language	Screening	1) Speech-language pathologist and CHW/NI 2) NI/10–15 min 3) CHW/9 min	Adapted as Application	1) Maleka, et al. 2016/; 2) du Toit, et al. 2021 3) Fuchs, et al. 2023
PEDS Developmental Milestones (PEDS:DM)	Expressive and Receptive Language	Screening	1) Speech-language pathologist and CHW/NI 2) NI/10–15 min 3) CHW/9 min	Adapted as Application	1) Maleka, et al. 2016/; 2) du Toit, et al. 2021 3) Fuchs, et al. 2023
Receptive-Expressive Emergent Language Test – Third Edition (REEL-3) Screening Kit of Language and Development (SKOLD)	Receptive and Expressive Language, Play/Social Behavior	Screening	Speech-language pathology undergraduates/25 min	Adapted for Synchronous Teleconsultation (use of traditional printed material)	Ciccia, et al. 2011
Web-based Clinical Decision Support System (CDSS)	Sensory Reception, Speech Perception, Speech Production, and Pragmatics	Screening	Teachers + Speech-language pathologists/NI	Developed for Teleconsultation – Online Platform	Ruiz, et al. 2014
Story retelling, analysis, NWR, GSI, and parental questionnaire	Phonological Processing, Narrative Organization, and Grammatical Competence	Screening	Speech-language pathology graduate students/NI	Synchronous and Asynchronous Teleconsultations (video analysis without a specific protocol)	Guiberson, et al. 2015
Video-recorded behavioral measures and parent reports on vocabulary	Gesture Typology; Symbolic Play; MLU-W; NDW	Screening	Screening facilitated by a speech-language pathologist or native Spanish-speaking educator, with video analysis conducted by ten graduate research assistants/NI	Synchronous and Asynchronous Teleconsultations (video analysis without a specific protocol)	Guiberson, et al. 2016
PLS-4 Screening Test	Language Sound Production (articulation/phonology/inconsistency)	Screening	Speech-language pathologist/ HBT – 12.9 min SBT – 14.1 min	Synchronous Teleconsultation	Taylor, 2017
Telehealth Evaluation of Development for Infants (TEDI)	Vocalizations, verbalizations, and gestures	Screening	NI	Synchronous/Asynchronous Teleconsultation (interaction video analysis)	Talbott, et al. 2020
Language Screen	Receptive and Expressive Vocabulary Sentence Repetition Auditory Comprehension	Screening	Teachers and assistants/10 min	Developed for Telehealth (App and Website)	Hulme, et al. 2024
SRESHT Screener	Receptive and Expressive Language	Screening	CHWs and speech-language pathologist/NI	Developed for Telehealth (Application)	Varadharajan, et al. 2024
Peabody Picture Vocabulary Test, Revised (PPVT-R)	Receptive Vocabulary	Assessment	Speech-language pathologist/NI	Synchronous Teleconsultation	Haaf, et al. 1999
Peabody Picture Vocabulary Test-3rd edition (PPVT-III); Preschool Language Scale-4th Edition (PLS-4)	Expressive and Receptive Language	Assessment	Speech-language pathologist/NI	Synchronous Teleconsultation	Eriks-Brophy, et al. 2008
CELF – Fourth Edition (CELF-4)	Receptive and Expressive Language	Assessment	Speech-language pathologist/30–60 min	Adapted for Synchronous Teleconsultation with digitized test	Waite, et al. 2010
Peabody Picture Vocabulary Test – Fourth Edition (PPVT-4)	Receptive Vocabulary	Assessment	NI/11.1 min	Adapted for Synchronous Teleconsultation – Online Platform	Anderson, 2014
MacArthur–Bates Communicative Development Inventories (CDIs)	Receptive and Expressive Vocabulary	Assessment	Caregivers/5.1 min	Adapted for Asynchronous Teleconsultation (Online Platform)	1) Makransky, et al. 2016 2) Kachergis, et al. 2022
MacArthur–Bates Communicative Development Inventories (CDIs)	Expressive Vocabulary; Receptive Vocabulary	Assessment	Caregivers/NI	Adapted for Asynchronous Teleconsultation (Online Platform)	de Mayo, et al. 2021
Diagnostic Evaluation of Language Variation – Norm Referenced (DELV-NR) Comprehensive Receptive and Expressive Vocabulary Test – Third Edition (CREVT-3) Test of Language Development (Primary) – Fifth Edition (TOLDP:5)	Receptive and Expressive Vocabulary	Assessment	Speech-language pathologist/mean 32 min	Adapted for Synchronous Teleconsultation with digitized tests acquired from publishers	Schmitt, et al. 2022
CELF (Clinical Evaluation of Language Fundamentals)	Receptive and Expressive Language	Assessment	Speech-language pathologist/NI	Synchronous Teleconsultation	Campbell, et al. 2024

Legend: min = minutes; NI = not informed; MLU-W = Mean Length of Utterance in Words; NDW = Number of Different Words produced; NWR = Nonword Repetition; GSI = Grammaticality Score Index

Source: Author

**Chart 1:** Descriptive synthesis of the findings of the included studies (n=20)

Author/ Year/ Location	Article Title	Study Type	Screening/ Assessment	Tool Used	Sample Size (N)	Objective	Comparison Measures	Results	Conclusions
Anderson/ 2014/ USA	Web-Based Telerehabilitation for Assessing Receptive Language in Children	Methodological validation study	Assessment	“Peabody Picture Vocabulary Test, Fourth Edition (PPVT-4) in web format.”	34 children aged between 4 and 13 years.	To evaluate the efficacy of a web-based receptive language test compared to the traditional test.	Remote and in-person administration of PPVT-4.	“The web test showed results comparable to traditional ones. There was no significant difference between the two administration formats (p > 0.05).”	Telehealth can be a viable alternative for language assessment in underserved populations.
Ciccia et al./ 2011/ USA	“Improving the access of young urban children to speech, language and hearing screening via telehealth”	Exploratory feasibility study	Screening	“Receptive-Expressive Emergent Language Test – Third Edition (REEL-3): This test was used for children up to three years old or with estimated language skills in that age range and the Screening Kit of Language and Development (SKOLD): for children 2.5 to 4 years old, testing expressive and receptive language. The Preschool Language Scale 4 Articulation Screener (PLS-4) test was applied to children who underwent assessment with the SKOLD, to verify the phonetic inventory.”	“411 screenings over 24 months of study (263 in language screening). In the first year, 33% of the children were up to three years old (n=60) and in the second year, 40% (n=91).”	“To analyze the feasibility of using low-cost videoconferencing in urban community health clinics for speech, language, and hearing screening in children up to six years of age, as well as to assess family satisfaction with videoconferencing and the reliability of remote screenings.”	A comparison was made between the pass and fail rates of screening tests applied in-person and via videoconferencing (Skype).	“High family satisfaction rate, 72% scheduled complete evaluations after screening failures. 100% reliability for hearing and language screenings.”	“The use of teleconsultations can improve access to screening services for urban families, overcoming barriers to healthcare access.”
Guiberson et al/ 2015/ USA	Accuracy of Telehealth-Administered Measures to Screen Language in Spanish-Speaking Preschoolers	Methodological validation study	Screening	Questionnaire with parents; and video recording of a story retelling sample for analysis of Nonword Repetition (NWR) and agrammatism index (AI).	“82 children between 37 and 69 months of age (mean = 53.65 months)”	To evaluate the accuracy of telehealth-administered language screening measures to Spanish-speaking children.	“The results of the screening test battery were compared with the SPLS-4 (Spanish Preschool Language Scale, 4th edition)”	“NWR scores, agrammatism index, and SDLQ measures were significantly correlated with the standard expressive language scores of the SPLS-4 (r = 0.55, p < 0.01; r = −0.38, p < 0.01; vocabulary, r = 0.46, p = 0.01; language questions, r = 0.45, p = 0.01; M3L-W, r = 0.42, p = 0.01). However, only when NWR scores were combined with language sample measures or parent survey, promising classification accuracy values that approached or were greater than 0.8 were obtained.”	“A hybrid telehealth model may be effective in screening for language development in Spanish-speaking children, especially when processing efficiency measures are combined with parent questionnaires or language samples.”
Guiberson/ 2016/ USA	Telehealth Measures Screening for Developmental Language Disorders in Spanish-Speaking Toddlers	Methodological validation study	Screening	Video-recorded behavior measures and parent reports on vocabulary.	“62 children between 2 and 2.11 years old and their mothers”	To evaluate the efficacy of a hybrid model for screening developmental language in Hispanic toddlers.	“The results of the screening test battery were compared with the SPLS-4 (Spanish Preschool Language Scale, 4th edition).”	“Gesture and play measures were not significantly correlated with language scores on the Spanish expressive SPLS-4. However, both language sample measures (MLU-W and NDW), as well as parent report measures (reported vocabulary and RM3L) were significantly correlated (p≤0.01) with language scores. The results showed that improved classification precision and accuracy can be achieved when reported vocabulary is combined with NDW.”	Hybrid screening models for the studied population are promising, offering a viable alternative for language screening.
Taylor/ 2017/ Australia	Speech and Language Screening for Children with Medical Complexity	“Two studies^(^1^)^: descriptive-exploratory (Survey), with speech-language pathologists, seeking to understand barriers for children with medical complexity (CMC)* to access speech-language pathology services and the adoption of telehealth in language screening in this population^(^2^)^; Methodological validation study”	Screening	For language screening, the following tests were used: PLS-4 Screening Test (3 to 5 years) or CELF-4 Screener (6 to 12 years).	(1) Survey applied at two times (n=46) in 2011 and (n=47) in 2015^(^2^)^, 34 children aged between 3 and 12 years	To evaluate the efficacy of speech and language screening in children with medical complexity.	“A comparison was made between screening tests applied in-person and in a remote setting using two methods, the Hardware-based telepractice (HBT) and System Software-based telepractice (SBT) System using consumer-grade equipment.”	“The correlation was moderate for the PLS-4 screening test with the HBT method (r = 0.39) and for the CELF-4 screener with the SBT method (r = 0.44), while it was high for the PLS-4 screening test with the SBT method (r = 0.65) and for the CELF-4 screener with the HBT method (r = 0.68).”	Screening is a valuable tool for improving access to speech-language pathology services for children with medical complexity.
Campbell et al./ 2024 / USA	Reliability and Feasibility of Administering a Child Language Assessment via Telehealth	Methodological validation study	Assessment	CELF (Clinical Evaluation of Language Fundamentals). Depending on the age, the CELF Preschool–Third Edition; CELF 5, 5 to 8-year-old version; CELF-5, 9 to 21-year-old version	100 children aged 3 to 12 years	To evaluate the efficacy of remote administration of child language assessments, CELF (Clinical Evaluation of Language Fundamentals) in in-person and remote format	CELF (Clinical Evaluation of Language Fundamentals), administered in both in-person and remote formats.	“Bland-Altman analysis, paired t-tests, there were no significant differences in standard scores between administration conditions.”	“Remote administration of language assessments is feasible and reliable, with no significant systematic differences.”
“Hulme, et al/ 2024/ England”	“Language Screen: The Development, Validation, and Standardization of an Automated Language Assessment App”	Methodological validation study	Screening	Language Screen	350,000 children aged 3 years and 6 months up to 8 years and 11 months	“To develop and standardize the mobile application “Language Screen”, providing teachers and educational professionals with a fast and reliable method to assess children's language ability”	“A total of 1,156 children, who presented the lowest scores in their classes, were reassessed by speech-language pathologists using standardized measures (such as the Clinical Evaluation of Language Fundamentals (CELF), Expressive Vocabulary and Sentence Recall and the Renfrew Action Picture Test Information and Grammar). Total language assessment scores made by speech-language pathologists correlated strongly with total Language Screen scores (r = 0.74).” For the overall sample, psychometric analysis of the application was performed using the Rasch model and other statistical tools to evaluate its properties and validity.	“Language Screen presents excellent psychometric properties, including high reliability, good fit to the Rasch model, and minimal difference in item functioning across major student groups.”	“The Language Screen provides an easy-to-use and reliable method for identifying children with language difficulties. Its use in schools can help increase teacher awareness of variations in language skills and their importance for educational practice.”
“Kachergis, et al/ 2022/ USA”	Online Computerized Adaptive Tests of Children’s Vocabulary Development in English and Mexican Spanish	Methodological validation study	Assessment	CDI-CAT	251 children aged between 15 and 36 months	“To create computerized adaptive versions (CAT) of the CDIs, in English and Spanish.”	“There was a comparison between the results of the traditional versions, the MacArthur–Bates Communicative Development Inventories (CDIs), with the adaptive version (CDI-CAT).”	“The adaptive test proved effective in retrieving participants' ability with high correlation regarding estimates made with the complete test. The study found a high correlation (r = 0.92) between estimated abilities from the full test and the adaptive test.”	The adaptive version of the test offers a more efficient yet accurate way to assess the vocabulary of young children. The study provides recommendations for creating adaptive versions in new languages and discusses when this approach is suitable.
“Maleka, et al/ 2016/ South Africa”	Developmental Screening—Evaluation of an m-Health Version of the Parents’ Evaluation Developmental Status Tool	Methodological validation study	Screening	Parents’ Evaluation of Developmental Status (PEDS) and PEDS: Developmental Milestones (PEDS:DM)	“207 caregivers who were in a child welfare clinic. The caregivers were divided into a stratified sample according to their children's age groups. The age groups were 6 to 18 months (69%, n = 142) and 19 to 36 months (31%, n = 65).”	To evaluate developmental screening in terms of 1-correspondence between the conventional paper-based test performed by the speech-language pathologist and the test using a smartphone application performed by the community health worker, and 2- the inter-rater reliability between the speech-language pathologist and the community health worker.	Correspondence between test methods (paper and application) and Inter-rater reliability (speech-language pathologists and CHWs).	“A high positive (100%) and negative (96%) correspondence was found between the paper PEDS tools and the smartphone application, as well as between the SLP and the CHW. Near-perfect inter-rater agreement (Cohen’s Kappa) was demonstrated between conditions (κ=0.873 to κ=0.961). “	“The smartphone application results, operated by a Community Health Worker (CHW), closely matched the standards of the PEDS tools operated by a health professional. Trained CHWs can perform accurate developmental screenings using the smartphone version of the PEDS tools.”
“Ruiz, et al/ 2014/ Spain”	Evaluating a Web-Based Clinical Decision Support System for Language Disorders Screening in a Nursery School	Methodological validation study	Screening	Web-based Clinical Decision Support System (CDSS) - online platform	“146 children, 94 from 0 to 3 years and 52” in the 4- to 6-year-old phase	“The study evaluates a system called CDSS, which is a digital tool designed to aid in the screening of language disorders in young children, especially in settings like nursery schools.”	A comparison of the results provided by the system and the evaluation of a speech-language pathologist was performed, however, there was no detail on this evaluation.	“The validation process showed an overall success rate of 83.6% (122/146) in language assessment and a rate of 7% (7/94) of unaccepted system decisions within the 0- to 3-year-old age range. The system helped speech-language pathologists identify new children with possible disorders who needed further evaluation.”	“This research demonstrated the benefit of the web-based CDSS for monitoring child neurodevelopment through the early detection of language delays in a nursery school. The system helped identify children with possible language disorders who needed additional assessment. However, the system needs adjustments, as therapists suggested changes to the knowledge base, including new questions about speech production and pragmatic skills.”
“Talbott, et al./ 2020/ USA”	Brief Report: Preliminary Feasibility of the TEDI: A Novel Parent - Administered Telehealth Assessment for Autism Spectrum Disorder Symptoms in the First Year of Life	Exploratory feasibility study	Screening	“Telehealth Evaluation of Development for Infants (TEDI) - during teleconsultations, parents perform play activities with their babies following the instructions on the interaction cards and the materials provided in the TEDI kit. These sessions are video recorded for later analysis. Tests used: CSBS-DP Infant-Toddler Checklist (ITC); Ages and Stages Questionnaires: 3rd Edition (ASQ-3); Ages and Stages Questionnaire: Social Emotional, Second Edition (ASQ:SE-2); Autism Observation Scale for Infants (AOSI)”	Children between 6 and 12 months and their parents	“To investigate the feasibility of a novel Level 2 telehealth assessment of infants' early social communication and ASD symptoms, the Telehealth Evaluation of Development for Infants (TEDI).”	Comparisons were made between the results of different evaluators and between test and retest.	“Infants averaged 10.23 months at the time of the TEDI 1a visit (with a range of 6.30 to 14.00 months). TEDI 1a and 1b sessions occurred, on average, 22 days apart (ranging from 7 to 41 days). As expected, there were significant associations between infants’ age (in months) at TEDI 1a and all three behavioral measures (AOSI total score: rs​=−0.64,p=0.034; AOSI markers: rs​=−0.82,p=0.002; ECI: rs​=0.73, p=0.02)”	“The study highlights the feasibility of the adapted telehealth protocol for behavioral screening of infants with ASD symptoms, being the first to systematically test this approach. The results indicate that the protocol is acceptable to parents and may lead to earlier referrals and better outcomes for infants with persistent symptoms. However, the lack of diagnostic data and the limited sample size are significant limitations, and more research with larger samples is needed to validate the results and evaluate the clinical usefulness of the telehealth screening protocol.”
“Varadharajan, et al/ 2024/ India”	Efficacy of a Mobile Health Screening Tool for Developmental and Speech Language Disorders	Methodological validation study	Screening	“SRESHT screener, a tool developed by the group for developmental and language screening.”	“9 Community Health Workers (CHWs), 1 speech-language pathologist who validated the screening applied by the CHWs in 109 children aged 0 to 6 years.”	To evaluate the efficacy of the SRESHT screener in screening children for speech and language disorders.	No comparison was made with in-person measures. Only inter-rater reliability analysis was performed.	“Analysis of the RBSK developmental screening results indicated a “"substantial agreement”” (κ=0.62), according to Cohen's kappa coefficient. Furthermore, a “"near-perfect agreement”” (0.81) was observed in the comparison between the screening results performed by the SLP and the GRW.”	The use of technology can improve the screening of speech and language disorders in rural communities.
“Waite, et al./ 2010/ Australia”	Internet-Based Telehealth Assessment of Language Using the CELF–4	Methodological validation study	Assessment	CELF–4 (Clinical Evaluation of Language Fundamentals).	“25 children (4 aged 5 years, 8 aged 6 years, 3 aged 7 years, and 10 aged 8 years) and 3 speech-language pathologists.”	To examine the validity and reliability of the videoconferencing system for assessing the core components of a standardized assessment tool.	Comparison of intra- and inter-rater administration of the CELF-4 in-person and remotely.	“High intra- and inter-rater reliability was observed, with Intraclass Correlation Coefficient ranging from 0.84 to 1.00 for different measures, with 92% total agreement among participating speech-language pathologists.”	The CELF-4 test is valid and reliable for remote administration by a speech-language pathologist.
“Schmitt, et al/ 2022/ USA”	Feasibility of Assessing Expressive and Receptive Vocabulary via Telepractice for Early Elementary-Age Children With Language Impairment.	Methodological validation study	Assessment	Diagnostic Evaluation of Language Variation–Norm Referenced (DELV-NR); Comprehensive-Receptive and Expressive Vocabulary Test–Third Edition (CREVT-3); Test of Language Development (Primary)–Fifth Edition (TOLDP:5).	20 children with a mean age of 5 years and 11 months	To investigate the procedures necessary to perform receptive and expressive vocabulary assessments via telepractice in children with intellectual disability and to evaluate the reliability of scores from these virtually administered assessments.	No comparison was made with in-person measures. Only inter-rater reliability analysis was performed.	“All ICCs were strong for each of the three norm-referenced measures across all subtests, ranging from 0.937 to 0.998. The results suggest that these adaptations result in strong inter-rater reliability for scoring participants' responses in an online format.”	The study results suggest that conducting telepractice assessments can be a useful and reliable tool for school speech-language pathologists.
“Fuchs, et al/ 2023/ South Africa”	“Combined screening of early childhood development, hearing and vision by community health workers using mHealth tools in a low-income-community”	Descriptive-analytical observational study	Screening	“The mHealth developmental screening consisted of the Parents' Evaluation of Developmental Status (PEDS) and PEDS Developmental Milestones (PEDS: DM) tools; hearing screening - hear Screen; vision screening - Vula Vision application”	“63 children between 4 years and 1 month and 6 years and 11 months, with a mean age of 61.78 months (SD 6.131).”	To describe a combined screening program (developmental and sensory – hearing and vision) supported by mHealth conducted by community health workers with children aged four to six years (n = 63) in early childhood development centers in a low-income community	No comparison was made with in-person measures. Only the description of referral rates was performed.	“The overall referral rate for developmental screening was 30% (n = 19), for hearing screening was 6% (n = 4), and for vision screening was 5% (n = 3). The majority of children (64%; n = 40) passed all three screening tests. No child was referred in both sensory screenings. One child (2%; n = 1) was referred in both the developmental and vision screenings, and two children (3%; n = 2) were referred in both the developmental and hearing screenings. Some children (25%; n = 16) were referred only in the developmental screening, two children (3%; n = 2) were referred only in the vision screening, and two children (3%; n = 2) were referred only in the hearing screening. The referral rate for developmental screening was significantly higher (30%) compared to the referral rate for vision (5%; z=4.183, p<0.001) and hearing (6%; z=3.908,p<0.001), respectively.”	“The integrated screening program, supported by mHealth and conducted by community health workers along with child development professionals, proved to be feasible, contributing to the early detection of developmental delays and sensory losses in children from low-income communities.”
“Makransky, et al/ 2016/ USA”	“An Item Response Theory–Based, Computerized Adaptive Testing Version of the MacArthur–Bates Communicative Development Inventory: Words & Sentences (CDI:WS)”	Methodological validation study	Assessment	CDI-CAT	1,461 children aged between 16 and 30 months	“To investigate the feasibility and potential validity of a computerized adaptive testing (CAT) version, aiming to reduce the length while maintaining measurement precision.”	A comparison was made between the results of the complete CDI with the adaptive version (CDI-CAT).	“Simulations of real CDI-CAT data with at least 50 items presented correlations above 0.95 compared to the full CDI results. Furthermore, these CDI-CATs showed similar correlations with age and socioeconomic status compared to the full CDI:WS.”	The results indicated strong evidence that a CAT version of the CDI:WS has the potential to reduce length without compromising the accuracy and precision of the complete instrument.
“deMayo, et al. / 2021/ USA”	Web-CDI: A system for online administration of the MacArthur Bates Communicative Development Inventories	Descriptive-analytical observational study	Assessment	Web-CDI	“*CDI Words & Gestures (WG) - 1620 forms, *CDI Words & Sentences (WS) - 1900 forms”	“To present Web-CDI, a web-based tool that allows researchers to collect CDI data online.”	No comparison was made with in-person measures. Only a general comparison was made between the demographic characteristics obtained in previous works using the paper-and-pencil CDI form with those obtained by Web-CDI	“More than 3,500 valid administrations of the WG and WS forms were collected on Web-CDI by more than a dozen researchers in the United States, after applying strict exclusion criteria derived from previous normative studies. The demographic trends observed in previous works using the paper-and-pencil CDI form were replicated in the data obtained by Web-CDI.”	Web-CDI is a valid alternative to the paper form and captures similar results
“du Toit, et al/ 2021/ South Africa”	mHealth developmental screening for preschool children in low-income communities	Methodological validation study	Screening	mHealth Parents’ Evaluation of Developmental Status (PEDS) and PEDS: Developmental Milestones (PEDS: DM)	276 participants with children between three years and six years and 11 months	To validate an mHealth-based developmental screening tool for children aged 36 to 83 months compared to a standardized developmental assessment instrument. It also sought to determine whether a significant association exists between referral criteria.	“A comparison was made between the screening results with the mHealth PEDS tool and the results obtained with the Vineland Adaptive Behavior Scales, Third Edition (Vineland-3).”	“The mHealth PEDS tools identified 237 children (85.9%) at risk for developmental delay, while the Vineland-3 identified 221 children (80.1%) with delay. The sensitivity of the PEDS tools was high (92.6%), but the specificity was low (22.5%) when using standardized US criteria. According to the PEDS: DM (89.3%; n = 142) and the Vineland-3 (87.1%; n = 134), literacy skills were the most delayed.”	“Despite the mHealth PEDS tools presenting high sensitivity and lower specificity, they may be useful for monitoring child development in preschool children in low-income settings.”
“Eriks ‐ Brophy, et al. /2008/ Canada”	Part of the problem or part of the solution? Communication assessments of Aboriginal children residing in remote communities using videoconferencing	Exploratory and reliability study	Assessment	“Peabody Picture Vocabulary Test-3rd edition (PPVT-III); Preschool Language Scale-4th edition (PLS-4); subtests of the Clinical Evaluation of Language Fundamentals-4th edition (CELF-4); Expressive One Word Picture Vocabulary Test (EOWPVT), Goldman Fristoe Test of Articulation-2nd edition (GFTA-2)”	7 children aged 4;3–12;9. These children were referred for assessment by caregivers or teachers due to concerns regarding their speech and/or language development.	“To examine how the issue of test bias may affect the use of videoconferencing in speech-language pathology assessment for northern and Aboriginal communities in Ontario, complemented by the collection of community perceptions on the usefulness of these technologies.”	A percentage agreement analysis was performed between the two speech-language pathologists responsible for filling out the tests, one present on-site and the other working remotely.	“Percentage agreement ranged from 96% to 100% for language tests and from 66% to 100% for the articulation measure. The children generally adjusted well to the system, responding easily to the remote speech-language pathologist and following test instructions. However, all required an adjustment period to understand the test dynamics and how to interact with the technology.”	The results suggest that videoconferencing can be an effective complement to service delivery when procedures are organized to minimize bias in test administration and interpretation of test performance.
“Haaf, et al/ 1999/ Canada”	Computer-Based Language Assessment Software: The Effects of Presentation and Response Format	Experimental study	Assessment	“Peabody Picture Vocabulary Test, Revised (PPVT-R)”	72 children with normal development between the ages of 4:0 (years:months) and 8:11	“To investigate the effects of computerized presentation of the PPVT, Form M, using two computer-based response formats.”	A comparison was made between the results of the three testing conditions (standard, computer with trackball, and with automated scanning).	“The main effects for testing condition (F = 0.217, p = 0.81) and age (F = 0.46, p > 0.50) were not significant, and there was no significant interaction between these factors (F = 0.491, p > 0.61). These results indicated that there was no significant difference in subject performance across the three testing conditions, and that test performance in each condition did not vary significantly as a function of age.”	The adapted response formats of the computerized version constitute statistically equivalent forms of the PPVT-R and can be used with the published norms for this test.

* (CMC) = group of children with one or more chronic and severe medical conditions, functional limitations, and extensive service needs

Caption: HBT = Hardware-based telepractice; SBT = Software-based telepractice

Source: Prepared by the author.

Chart 1 presents general information, including authors, year, study location, article title, study design, objective, focus on screening or assessment, sample size, children’s age, tools used for screening or assessment of language, main results, and conclusions. Additionally, Chart 2 details the characteristics and specificities of the protocols adopted by the authors, including language skills assessed, type of protocol or test (caregiver questionnaire or direct child observation), existence of prior validation in conventional format, professional training required for administration, availability of accuracy or validation values, method of administration (videoconference, online platform, or application), duration, and context of application (clinical or academic).

**Chart 2:** Specific characteristics of language screening/assessment protocols applied through digital health resources (n=20)

Name/Year/ Author	Target Skills	Test Type	Test Already Validated in In-Person Context (Y/N)	Professional's Background	Accuracy/Validation Data	Digital Application Method	Application Duration	Application Context
Anderson/ 2014/ USA	Receptive vocabulary	Direct observation of the child	Yes	NI	Criterion validity - Spearman's rank-order correlations revealed a strong positive correlation between the two sets of raw scores, “(r = 0.86).”	The test was administered via web browser. The clinician's and family's access was via computer.	11.1 minutes	Clinical
Ciccia et al./ 2011/ USA	“Sound production (articulation); expressive language, auditory receptive language, and play/social behavior. “	Parent questionnaire and direct observation of the child	Yes	Undergraduate students (15 weeks of application) supervised and trained	“No accuracy or validity tests were performed; only the reliability of the screening was investigated, which was determined by comparing the pass and fail rates for all screening components between those conducted via videoconference and those conducted in-person. In the first year of the study, the pass rate on language screenings was 72% of the total sample, while in the second year it was 61%.”	Teleconsultation via videoconference, “Computer kiosks were installed in the two clinics, each equipped with Dell laptops with 43 cm screens, webcams (Microsoft Lifecam VX-3000), and Skype 3.8 for Windows”	25 minutes for the complete screening (hearing, language, and speech)	Clinical
Guiberson et al/ 2015/ USA	“Phonological processing, narrative organization, and grammatical competence”	Direct observation of the child	No	Five postgraduate speech-language pathology students	“Accuracy and criterion validity data were established. The Nonword Repetition Test (NRT) presented an Area Under the Curve of 0.83, with sensitivity of 0.74 and specificity of 0.75. Positive and negative likelihood ratios were 2.94 (95% CI: 1.73–5.0) and 0.35 (95% CI: 0.20–0.63), respectively. The index of agrammatism obtained values of 0.68 for the Area Under the Curve, 0.59 for sensitivity, 0.67 for specificity, 1.76 (95% CI: 1.08–2.88) for the positive likelihood ratio, and 0.62 (95% CI: 0.39–0.97) for the negative likelihood ratio. The MLU-W (translation: Mean Length of Utterance - Words) presented an Area Under the Curve of 0.75, sensitivity of 0.66, specificity of 0.91, positive likelihood ratio of 7.70 (95% CI: 2.56–23), and negative likelihood ratio of 0.37 (95% CI: 0.25–0.56).The NRT and MLU-W measures demonstrated good concurrent validity.The RNP scores showed a significant correlation with the SPLS-4 expressive language standard scores (r = 0.55, p < 0.01). The agrammaticality index demonstrated a practically and statistically significant association with the SPLS-4 expressive language scores (r = −0.38, p < 0.01). A large effect size was observed for the association involving RPP, and a medium effect size was observed for the agrammaticality index, suggesting that these measures may be useful in language screening sessions delivered via telemedicine.”	The procedures were conducted using synchronous videoconferencing, e-book videocasting, and video recording. An iPad and a Flip video camera were used for data collection	NI	clinical
Guiberson/ 2016/ USA	“Gesture typology (deictic, iconic, and conventional); symbolic play; complexity of symbolic play, MLU-W (translation: Mean Length of Utterance - Words); NDW (translation: number of different words) produced”	Direct observation of the child	Yes	The screening process was facilitated by a speech-language pathologist, or a Spanish-speaking native educator, *Ten postgraduate research assistants performed the video analysis	“Accuracy was calculated only for the most promising individual screening measures. For the NDW measure (translation: number of Different Words), the Area Under the Curve was 0.82, sensitivity was 0.73, specificity was 0.83, the positive likelihood ratio (±LR) was 4.16 (95% CI: 2.02–8.54), and the negative likelihood ratio (−LR) was 0.33 (95% CI: 0.17–0.66), demonstrating suggestive values. Furthermore, the same values were calculated for the association of the NDW measure with the information obtained in the questionnaire. Results: sensitivity of 0.86 and specificity of 0.93. The (± LR) was 11.51 (95% CI: 3.83–34.63), while the (-LR) was 0.15 (95% CI: 0.05–0.42). These combined measures increased the accuracy of the screening.Convergent validity: Gesture and play measures were not significantly correlated with language scores on the expressive Spanish SPLS-4. However, both language sample measures, MLU-W and NDW, were significantly correlated (p≤0.01) with the language scores of the gold standard test”	"Asynchronous videoconferencing—Video recordings were conducted in the child’s home or in clinical centers. During these recordings, children and parents interacted with preselected toys. The recordings were captured using a mini video camera with a sampling rate of 48 kHz."	NI	clinical
Taylor/2017/ Australia	Language; Sound production (articulation/phonology/inconsistency); Oromotor Assessment (assessment of sound sequence production and of isolated and sequenced oral movements.	Direct observation of the child	Yes	Speech-language pathologist	“There was high agreement between in-person scores and scores obtained by HBT (Hardware-based telepractice) for inconsistency and oromotor tasks. Agreement was also high between in-person scores and scores obtained by SBT (Software-based telepractice) for oromotor screening and the PLS-4 Screening Test. However, validity was reduced for some screening tasks and age groups, and several individual speech sounds could not be validly screened using telepractice (all fricatives or high-frequency sounds). For preschool-aged children, the accuracy of the scores on the PLS4 Screening Test was high (large positive correlation) during SBT screening and only medium for HBT screening.”	Synchronous Video Conferencing"HBT (Hardware-based telepractice) system: the videoconference was conducted using Cisco TelePresence System, Quick Set C20 systems, *SBT (Software-based telepractice) - videoconference was conducted between two laptops (Dell Vostro 3550), with external webcams (Logitech QuickCam Pro 9000) using free videoconferencing software (Logitech Vid HD, Cisco Jabber Video for Telepresence)"	HBT: 12.9 minutes; SBT: 14.1 minutes.	clinical
Campbell et al. / 2024 / USA	Receptive and expressive language, “From 3 to 5 years old, the following subtests were applied: Sentence comprehension, word structure, and expressive vocabulary.”, “From 5 to 8 years old, the following subtests were applied: sentence comprehension, word structure, sentence formulation, and sentence recall.”, “From 9 to 12 years old, the following subtests were applied: word classes, sentence formulation, sentence retelling, and semantic relationships.”	Direct observation of the child	Yes	Five speech-language pathologists trained to assess language	“Mean Bias: The average of the standard scores from all CELF versions had a mean bias of 0, indicating no systematic difference between administration conditions. The skewness of the standard scores of the mean CELF differences ranged from -0.08 to 0.11, indicating a normal distribution Paired t-tests: there were no significant differences in standard scores between conditions, with small effect sizes. Bland-Altman analysis showed that the red line of equality (mean bias) fell within the 95% confidence interval, indicating no significant difference between conditions.”	Videoconferencing was conducted using Zoom for Healthcare (Zoom Video Communications, Inc., 2023) in a teleconsultation model. Speech-language pathologists used the following devices: MacBook, Lenovo desktop computers, Microsoft Surface Pro, iPad, and Dell laptops, whereas children primarily used tablets and smartphones. The sessions were not recorded.	NI	Clinical
“Hulme, et al/ 2024/ England”	Expressive Vocabulary; Receptive Vocabulary; Sentence Repetition; Auditory Comprehension	Direct observation of the child	No	Teachers and assistants	No accuracy measures were taken. The Language Screen presented good to excellent reliability for the total scale (ω = 0.92, ωh​ = 0.75). The hierarchical omega value indicates that 75% of the composite score variance is attributable to the variance of the general factor of the bifactor model. Classical reliabilities (alphas) for the separate subscales were adequate to good (EV = 0.79, VR = 0.74, SR = 0.79, LC = 0.80). The test-retest reliability of the total Language Screen score was assessed using a sample of children in reception classes in the UK (i.e., the first year of compulsory schooling). Test-retest reliability was good (r = 0.78, N = 9,778).	Application and website, “The user creates an account, enters the children’s data (name, gender, date of birth) and downloads a set of QR codes. The application is installed on an Android or Apple device and, to start the assessment, the examiner scans the QR code to identify the child.	10 minutes	School
“Kachergis, et al/ 2022/ USA”	Expressive vocabulary; receptive vocabulary	Parent questionnaire	Yes	The questionnaire is completed by the caregivers themselves on the online platform. The result guides them about the need for other assessments.	"Comparisons were made between the estimated latent ability (θ) of the children from the CDI-CAT and the ability estimated via Item Response Theory (IRT) from the full CDI. The results show a strong correlation between the ability scores estimated by the CDI-CAT and the full CDI (r = 0.92). A strong correlation was also observed between the total vocabulary estimated from the full CDI and the ability estimated by the CDI-CAT (r = 0.86). The mean squared error between the abilities estimated by the CDI-CAT and the full CDI was 0.55, which suggests a good correspondence between the two methods. The adapted test also presented a high test-retest reliability index (0.95).	“Online platform. Caregivers are presented with one word at a time and must select “Yes” or “No” to indicate whether the child can say or understand that word.”	5,1 minutes	clinical
“Maleka, et al/ 2016/ South Africa”	“Expressive language, receptive language”	Parent questionnaire	Yes	Speech-language pathologist and Community Health Worker	“In the comparison between smartphone-based and paper-based methods (n = 105), positive agreement reached 100%, negative 96%, and overall, 100%. Between assessments made using two different smartphones (n = 102), positive agreement was 98%, negative 98%, and overall 100%. Negative agreement in the 19 to 36-month age range presented a more significant variation, with a rate of 88%, suggesting the need for more careful analysis in this specific age range. “	Application: “The PEDS tools were developed into a smartphone application, using the same algorithm as the conventional paper-based PEDS tools. Two Samsung Neo Trend smartphones (Android OS 4.4.1) were used to install the PEDS tool application. Each device was handled by a speech-language pathologist (SLP) and a community health worker (CHW).”	NI	Clinical
“Ruiz, et al/ 2014/ Spain”	Sensory Reception, Speech Perception, Speech Production, and Pragmatics.	Teacher questionnaire, based on the Denver Developmental Screening Test	Yes	Teachers and Speech-language pathologist	The Nursery School Language Therapist -NSLT accepted the decisions of the GADES Software in 93% (87/94) of cases from 0 to 3 years and 67% (35/52) in the 4-to-6-year stage. The comparison of results led to a total accuracy rate for the GADES Decision System of 84% (122/146). A total of 24 cases out of the sample of 146 defined a non-acceptance rate of 16% to be reduced with further refinement of the system.	Online Platform: “GADES Software - based on an ontology that organizes information about the child's language acquisition according to their age. The system includes more than 100 rules to generate alerts and/or alarms whenever deviations from the expected developmental stage for the child are detected.”	NI	School
“Talbott, et al./2020/ USA”	“Infants' communication and language ability, including vocalizations, verbalizations, and gestures.”,”Development of social and behavioral skills, such as social communication.”	Questionnaire and direct observation of the child	Yes	NI	“Analyses showed significant associations between infants' age and behavioral measures. The total AOSI score had a negative correlation with infants' age. (rs​=−0.64,p=.034), indicating that older infants had lower scores. The number of AOSI markers also had a negative correlation with age (rs​=−0.82,p=.002). On the other hand, the ECI score had a positive correlation with age (rs​=0.73,p=.02), suggesting that older infants had better communication outcomes.”	"Synchronous videoconferencing with interaction video analysis"	NI	clinical
“Varadharajan, et al/ 2024/ India”	“Motor, cognitive, social, language development. In language, it assesses - Receptive Language: comprehension of instructions, word and phrase recognition and Expressive Language: Word and phrase production, ability to appropriately use vocabulary and use of sentences to express ideas.”	Direct observation of the child	Yes (RBSK), Yes (LEST)	Community Health Worker and Speech-language pathologist	Not presented in the article, only agreement data (RBSK: κ=0.62 and LEST: κ=0.68)	SRESHT Application, for mobile devices (tablets, smartphones), which contains the already validated protocols for in-person application by health professionals. In the application, the CHW already received the pass/fail result, referring to a complete assessment.	NI	Clinical
“Waite, et al./ 2010/ Australia”	“The following subtests were selected: concepts and following directions, word structure, sentence repetition, sentence formulation for a comprehensive assessment of receptive and expressive language “	Direct observation of the child	Yes	Speech-language pathologist	Not presented in the article. Only the measurement of the intra- and inter-rater reliability index was performed.	Videoconferencing	Not specified, but could vary from 30 to 60 minutes	Clinical
“Schmitt, et al/ 2022/ USA”	Receptive and Expressive Vocabulary	Direct observation of the child	Yes	Speech-language pathologist	“Not informed, but the measures are validated to assess receptive and expressive vocabulary, in English”	Synchronous videoconferencing, “Zoom for Healthcare (Zoom Video Communications, Inc., 2023) in a teleconsultation model”	Average of 97 minutes (range of 70–127 min).	Clinical
“Fuchs, et al/ 2023/ South Africa”	“Expressive language, receptive language”	Questionnaire with Early Childhood Development professionals who accompanied the children in specialized centers	Yes	Community Health Worker	Not performed	Application, “A Samsung A3 smartphone with the latest Android operating system was used, with the CHWs asking the questions to the early childhood development professionals. After completion, the algorithm was automatically applied in the smartphone application, generating the results, including referrals.”	Isolated development screening - 9 minutes, “The average time for combined screenings was 11.72 min (SD 3.45 min).”	Clinical
“Makransky, et al/ 2016/ USA”	Expressive vocabulary; receptive vocabulary	Parent questionnaire	Yes	The questionnaire is completed by the caregivers themselves on the online platform. The result guides them about the need for other assessments	“The 50-item CDI-CAT presented a correlation greater than 0.95 with the full test and a reliability coefficient of 0.968. Correlation and reliability values increased as the test length increased. “	“Online platform. Caregivers are presented with one word at a time and must select “Yes” or “No” to indicate whether the child can say or understand that word.”	NI	Clinical
“deMayo, et al. /2021/ USA”	Expressive vocabulary; receptive vocabulary	Parent questionnaire	Yes	The questionnaire is completed by the caregivers themselves on the online platform. The result guides them about the need for other assessments	Not performed.	“Online platform. Caregivers are presented with one word at a time and must select “Yes” or “No” to indicate whether the child can say or understand that word.”	NI	Clinical
“du Toit, et al./ 2021/ South Africa”	Expressive and receptive language	Parent questionnaire	Yes	NI	“Sensitivity, specificity, negative predictive value, and positive predictive value were calculated for different types of referral criteria. The sensitivity of the mHealth PEDS tools decreased with the increase in the number of developmental domains in the PEDS:DM, ranging from 92.6% for the prescribed criteria to 74.4% for RC2. On the other hand, specificity showed an increase, ranging from 22.5% to 46.4%, although it remained low. Across the different referral criteria (RC), the positive predictive value (PPV) remained high (82.8%–84.7%), with a lower negative predictive value (NPV) (31.3%–40.7%). Convergent validity was assessed by comparing the results of the PEDS and the PEDS:DM with the Vineland-3. A moderately positive association was observed between adaptive behavior and literacy skills in the tests.”	Smartphone Application	Between 10 and 15 minutes	Clinical
“Eriks ‐ Brophy, et al. /2008/ Canada”	Expressive and receptive language	Direct observation of the child	Yes	Speech-language pathologist	Accuracy and validity data were not calculated. Only the analysis of mean percent agreement between raters was performed.	Synchronous videoconferencing	NI	Clinical
“Haaf, et al/ 1999/ Canada”	Receptive vocabulary	Direct observation of the child	Yes	NI	Not performed. Only the degree of statistical equivalence between the results from the ANOVA test was calculated.	Software - Computer with trackball and automated digitization.	NI	Clinical

Caption: NI= Not Informed; NR= Not Performed

Source: Own Author

Three experts identified as having the highest scientific output on the topic were consulted during the scoping review process to indicate additional references. After analysis, one article was included for data extraction. Additionally, the reference lists of the previously included studies were examined, resulting in 11 studies with potential for inclusion. After a full-text review in phases 1 and 2, six studies were excluded because they focused exclusively on therapeutic interventions or targeted school-aged populations.

The analysis of data from the included studies was conducted descriptively and qualitatively.

## RESULTS

For better organization, the results are presented according to the following items: study selection, study characterization, screening and assessment instruments, instrument performance, skills assessed, context and methods, professionals involved, and digital health resources used.

### Study selection

**Figure 1:**
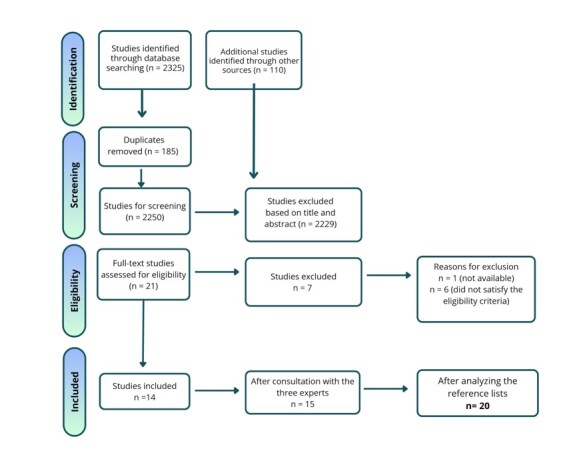
Review steps flowchart

The initial search identified 2,435 studies, of which 185 were excluded as duplicates. Of the remaining 2,250, 2,229 were eliminated during title and abstract screening due to misalignment with the review objectives (e.g., general approach to development, exclusive focus on speech, lack of primary data, or age incompatibility). After full-text analysis of the 21 remaining studies, seven were excluded, resulting in 14 articles. Additionally, inclusions were made via expert indication and review of the reference list, totaling 20 studies. The review stages are represented in the flowchart shown in Figure 1.

### Study characterization

The 20 included studies comprised mainly methodological validation research (n=14)^(45-58)^, followed by descriptive-analytical observational studies^(59,60)^, exploratory studies^(61-63)^, and experimental studies^(64)^. Five articles reported interrater reliability analysis^(48,53,55,56,62)^.

### Screening and assessment instruments

Seven assessment protocols (PPVT, CELF, CDI, PLS-4, CREVT-3, TOLDP:5, DELV-NR) and five screening tools (PEDS, PEDS:DM, PLS-4 screening, REEL-3, SKOLD), all of which are already used in face-to-face clinical practice, were analyzed in 14 of the 20 studies. The six remaining studies, focused on screening, were divided as follows: four developed digital tools for teleconsultation—LanguageScreen (application)^(51)^, SRESHT (application)^(48)^, Web-based Clinical Decision Support System - CDSS (online platform)^(54)^, and Telehealth Evaluation of Development for Infants - TEDI (observational protocol)^(62)^ — while two employed unstructured observation^(46,47)^.

### Instrument performance

Regarding instruments already validated for face-to-face application, the following was observed:

- PPVT (versions: PPVT-R, PPVT-III, and PPVT-4): Equivalent results to face-to-face application, with high interrater agreement and mean duration of 11.1 minutes^(45,63,64)^;
- CELF and CELF-4: High intra/interrater reliability^(55)^ and results equivalent to face-to-face^(50)^;
- PLS-4: High interrater agreement^(63)^; the PLS-4 Screening Test strongly correlated with face-to-face methods via Software-Based Telepractice (SBT), using laptops (mean duration: 14.1 minutes)^(49)^;
- PEDS and PEDS:DM: High positive (100%) and negative (96%) agreement between face-to-face and remote methods, using a smartphone application, as well as almost perfect interrater agreement (speech-language pathologist and community health worker)^(53)^. However, results also showed high sensitivity but variable specificity^(58)^;
- CDIs: Online and adaptive versions (CDI-CAT) showed high correlation with the original test^(52,57)^;
- CREVT-3, TOLDP:5, DELV-NR: No comparison with face-to-face measures, analyzed only for interrater reliability (ICC). ICC values were high across all subtests, ranging from 0.937 to 0.998, demonstrating strong agreement among raters^(56)^;
- REEL-3, SKOLD: 100% reliability^(61)^. The study did not provide details.

As for other tools, the following was observed:

- LanguageScreen: Excellent psychometric properties, including high reliability^(51)^;
- SRESHT: “Almost perfect” interrater agreement (0.81)^(48)^;
- Web-based Clinical Decision Support System (CDSS): Overall success rate of 83.6%^(54)^;
- TEDI: For this protocol, the TEDI kit was developed, containing materials and instruction cards for parents, aimed at stimulating specific interactions that allowed scoring of the Autism Observation Scale for Infants (AOSI) and the Individual Developmental Growth Indices - Early Communication Index (ECI). Intraclass correlation coefficients indicated good to excellent interrater agreement for total ECI and AOSI scores. In test-retest reliability, the AOSI reached statistical significance^(62)^. However, these instruments are not yet validated.

### Skills assessed

The measures encompassed receptive/expressive language^(48-50,53,55,58,59,61,63)^, vocabulary^(45,51,52,56,57,60,64)^, speech comprehension and production^(54)^, vocalizations/gestures and symbolic play^(47,62)^, phonological processing, and morphosyntactic skills^(46)^.

### Context and methods

Regarding participants, countries, and context, the following was observed:

- Sampling: Eleven studies employed direct child observation^(45-51,55,56,63,64)^; two combined questionnaires and observation^(61,62)^; and seven were based on parent/early childhood professional/teacher reports^(52-54,57-60)^. Sample sizes ranged from 7 to 1,461 participants.
- Countries: USA (n=10)^(45-47,50,52,56,57,60-62)^, South Africa (n=3)^(53,58,59)^, Australia (n=2)^(49,55)^, Canada (n=2)^(63,64)^, Spain^(54)^, England^(51)^, and India^(48)^ (n=1 each). Publications occurred between 1999 and 2024.
- Context: 18 studies were conducted in clinical settings^(45-50,52,53,55-64)^ and two in school settings^(51,54)^. Application time ranged from 5.1 to 97 minutes (only nine studies reported application time).

### Professionals involved

Speech-language pathologists conducted most assessments; however, some studies reported tests being administered by teachers^(51,54)^ and community health workers (CHWs)^(48,53,59)^.

### Digital health resources

Synchronous (videoconference) or asynchronous teleconsultations predominated (n=9)^(46,47,49,50,55,56,61-63)^, followed by online platforms (n=5)^(45,52,54,57,60)^, applications (n=5)^(48,51,53,58,59)^, and software use (n=1)^(64)^. Devices included computers, tablets, and smartphones.

The detailed synthesis of the screening/assessment protocols is available in Charts 1 and 2. The data on instruments used via digital health for language screening and assessment are presented in Table 1.

## DISCUSSION

The discussion presents three main axes: (i) the analysis of digital oral language screening and assessment instruments, considering their characteristics, objectives, and validation processes; (ii) the exploration of digital health resources in screening and assessment, with emphasis on technological tools and platforms; and (iii) the feasibility and applicability of digital oral language screening and assessment instruments, highlighting challenges, benefits, and implications for clinical practice.

### Analysis of digital oral language screening and assessment instruments

Of the 20 studies analyzed, 14 consisted of validation studies^(45-58)^, focusing on criterion validity—eight directed toward screening^(46-49,51,53,54,58)^ and six toward assessment^(45,50,52,55-57)^. However, the validation process proved heterogeneous. Ten studies validated the instruments by comparing previously validated face-to-face tests with their remote application or by confronting the results with a gold standard test^(45-47,49-53,57,58)^, an essential approach for evaluating the validity of an instrument^(9)^.

In contrast, one study validated the instruments through comparison between screening results and an assessment conducted by a speech-language pathologist, without specifying which tests were applied or which statistical measures were used^(54)^, evidencing low methodological rigor, since detailing the statistical tests is essential for an instrument to be considered suitable for clinical use^(10,14)^. Additionally, three studies were limited to interrater reliability analysis, a complementary measure to validity in evaluating instrument quality^(48,55,56)^.

Six studies presented descriptive^(59,60)^, exploratory^(61-63)^, or experimental^(64)^ designs, providing qualitative information such as family satisfaction^(59,61)^ and precautions necessary when conducting videoconferences^(63)^. Such data may support strategies to ensure the feasibility and effectiveness of assessments; however, equally essential details, such as administration time, were reported in a limited way^(59,62,63)^.

Variation was observed in the linguistic skills assessed, with predominance of research dedicated to screening protocols^(48,49,51,53,58,59,61)^ and assessment protocols^(45,50,52,55-57,60,63,64)^ of receptive and expressive language, as well as vocabulary, evidencing the robustness of these measures to identify risks of language problems and comprehensively assess child oral language.

To execute the tests, adaptations were made, including the digitization of physical materials^(45,49,50,55,64)^ and the audio recording of words and sentences, to standardize the presentation in online environments^(45,55,64)^. In some studies, even without recordings, commands were standardized by adopting the same approach as in traditional formats^(50)^.

During procedures, children were instructed to use the mouse to select the image corresponding to the word heard^(64)^, to say the related number, to point—with the hand or mouse—to the corresponding figure, or to draw on the screen using the annotation tool, allowing raters to make the selection^(45,49,50)^. Additionally, some devices featured touch screens, enabling the child to directly touch the image of the heard word^(55)^.

The studies analyzed indicate that traditional oral language assessment and screening instruments, such as PPVT^(45,63,64)^, MacArthur-Bates CDI^(52,57)^, CELF^(50,55)^, PLS-4 Screening Test^(49)^, PEDS and PEDS:DM^(53,58)^, can be applied in digital contexts, yielding results comparable to those obtained face-to-face, as confirmed by statistical tests. This finding suggests the possibility of validating oral language screening and assessment instruments in Brazilian Portuguese for digital applications, building on instruments already validated in face-to-face contexts. The choice of these instruments by researchers was based on the high degree of clinician familiarity and the ease of adapting materials to digital format^(45,64)^.

In assessment, the CELF test and its more recent editions assess language more broadly, covering receptive and expressive domains. In contrast, the PPVT and CDIs assess receptive vocabulary and the combination of receptive and expressive vocabulary, respectively. However, because they are more comprehensive, these tests require longer administration time^(55)^, which may pose a significant challenge in telehealth contexts due to connection limitations^(25)^. Therefore, studies investigating the feasibility of administering these tests in multiple sessions are needed.

Connection quality is a critical factor and may pose a challenge even in screening tests, as observed with the PLS-4 Screening Test^(49)^. Although screening via laptops was completed in 14.1 minutes, image distortions were frequent and, even with results correlated to face-to-face methods, such issues affected family satisfaction with the method.

In screening, PEDS and PEDS:DM showed high agreement between remote and in-person conditions, although the low specificity^(58)^ may imply a risk of high false-positive rates. It should be noted, however, that the gold standard used, Vineland-3, was not validated for the South African context, which may compromise accuracy when applied in that population^(12)^.

It is noteworthy that adapting widely consolidated face-to-face tests to the digital environment provides greater confidence in the quality and effectiveness of the tools, as these instruments are already well-known and validated in traditional contexts. At the same time, such familiarity may facilitate adherence by speech-language pathologists and promote effective use of protocols in digital settings^(45,64)^.

In addition to adapting traditional instruments, four studies focused on the development of tools specifically for teleconsultation^(48,51,54,62)^. All were created for screening, including two applications—LanguageScreen^(51)^ and SRESHT^(48)^; one online platform—CDSS^(54)^; and one protocol based on caregiver-child interaction observation—TEDI^(62)^. However, the evidence remains limited, as only one study provided detailed data on correlation with face-to-face measures^(51)^, a crucial procedure for validation^(9)^. The other studies were restricted to reporting interrater reliability indices^(48,62)^, test-retest reliability^(62)^, or stated that validation was performed by a speech-language pathologist, without detailing procedures or results^(54)^.

Thus, the limitation of data regarding correlation with face-to-face measures suggests that gaps in validation persist. However, aspects such as the use of low-cost digital tools, the possibility of administration by professionals other than speech-language pathologists, and the inclusion of screenings carried out in school contexts may provide relevant contributions, provided future research deepens validation in digital health.

Among the tools analyzed, only the PPVT and CELF-4 have been translated and adapted into Brazilian Portuguese, although they are still exclusively in a face-to-face format^(16,18)^. This scenario highlights the importance of research in facilitating translation, adaptation, and validation of instruments for use in both face-to-face and digital health contexts^(13)^.

### Digital health resources in oral language screening and assessment

Four studies used exclusively mobile phones for oral language screening, via applications^(48,53,58,59)^. However, only one of these studies provided data showing correspondence with face-to-face assessments^(53)^. It is noteworthy that all applications were developed in English, with one study conducted in India and three in South Africa. The protocols used in these studies have not yet been translated or adapted into Brazilian Portuguese, highlighting the need for research to address this gap and expand access to screening, as well as applicability in different linguistic and cultural contexts.

Although mobile phones constitute the main means of internet access and may offer significant contributions, a notable disparity is observed in connection quality across countries. In low-income nations, 3G technology predominates, while access to 4G and 5G networks reaches only 52% and 4% of the population, respectively. In contrast, in high-income countries, 5G access reaches 84%, with greater coverage in Europe, followed by the Americas. This disparity is also reflected in fixed broadband speed, with data indicating that, in less developed countries, the speed is 25 times lower than in high-income economies^(26)^.

These inequalities may compromise both accessibility and effectiveness of telehealth, especially in synchronous videoconferences, the most frequently used digital method in the clinical studies analyzed. Technical problems such as audio and video delays may negatively impact the consultation experience^(24,49)^. Beyond connection quality limitations, additional challenges arise, such as the need for digital skills among participants and compatible equipment to ensure successful care^(27,28)^. Even so, videoconferencing remains the preferred digital connection method for patients and clinicians, as it enables real-time interaction with mutual visualization^(24)^.

In school contexts, the studies analyzed explored the use of applications and online platforms^(51,54)^, aimed at teacher-administered screening. Procedures were carried out using computers^(54)^ and mobile devices^(51)^, in countries such as Spain and England, respectively, with an application time of 10 minutes in one study^(51)^. Access to reliable and valid tools that promote time and cost savings is one of the challenges for implementing screening in school settings, even in developed countries^(15)^. Contributions in this area may improve understanding of the feasibility and effectiveness of digital screening, supporting the development of public policies that integrate this practice into educational contexts, facilitating early identification of language difficulties, and expanding access to specialized interventions by speech-language pathologists and interdisciplinary teams.

### Feasibility and applicability of digital oral language screening and assessment instruments

Relevant data for test feasibility, such as administration time, were reported in a limited way, appearing in only nine studies^(45,49,51,52,55,56,58,59,61)^. These data, together with information on psychometric quality, accessibility, instrument costs, and training requirements, are determining factors in test selection^(19)^.

Regarding the professionals who administered screenings, two studies compared results obtained by trained community health workers (CHWs) with those obtained by speech-language pathologists, both of whom used a smartphone application to apply the tests^(48,53)^. One of these studies referred to SRESHT^(48)^, developed specifically for the digital environment, while the other consisted of an adaptation of the PEDS and PEDS:DM tests^(53)^. Results demonstrated substantial to almost perfect agreement between methods, suggesting that trained CHWs can perform developmental screenings accurately, representing significant progress in qualifying healthcare and expanding oral language screening coverage—especially considering that the number of CHWs is considerably higher than that of speech-language pathologists, particularly in low- and middle-income countries^(6-8)^.

It is observed that most of the studies analyzed were concentrated in North America, especially in the United States, evidencing the predominance of this region in scientific production on the subject. Although with objectives distinct from this scoping review, two systematic reviews also highlighted the US leadership in research on telehealth in speech-language pathology^(40,41)^, reinforcing the need to expand such investigations in underdeveloped and developing countries, where the implementation of digital solutions may significantly impact access to health services.

On the other hand, no studies originating from Latin America, the Middle East, or East Asia were identified. For the application of these protocols in the Brazilian context, it is essential to validate instruments for Brazilian Portuguese, considering the target population and regional specificities, as well as social, linguistic, and educational contexts^(20)^. Furthermore, access to quality internet must be considered.

Therefore, future research should prioritize detailed reporting of these variables, allowing an in-depth analysis of the feasibility and applicability of oral language screening and assessment instruments in digital health, especially considering the access of vulnerable populations and regions with limited resources.

## CONCLUSION

Eleven studies investigating screening instruments and nine focused on oral language assessment in early childhood were identified.

The equivalence between telehealth-based and conventional face-to-face assessment results suggests that these technologies can be effectively integrated into speech-language pathology practices and, in the case of screening, by other healthcare professionals, provided that appropriate training and adherence to best practices in tele-speech-language pathology are ensured.

Although digital instruments for early childhood oral language screening and assessment have demonstrated reliability and applicability within their contexts, further validation is still needed, considering the linguistic and cultural contexts of different countries.

The analysis of this study reveals that expanding digital health for oral language screening and assessment in Brazil requires not only scientific advances but also comprehensive improvements. These include the implementation of public policies that ensure access to digital health, the provision of high-quality internet, and the adequate training of healthcare professionals and speech-language pathologists to deliver care using such technologies.

## Appendix 1 Literature search strategies

**Table:**

Database	Search (August 9th 2024)
Embase	#1. 'developmental language disorder'/exp OR 'language development disorder' OR 'language development disorders' OR 'developmental language disorder' OR 'communication disorder'/exp OR 'communication disease' OR 'communication disorders' OR 'communication problem' OR 'communicative disorder' OR 'social communication disorder' OR 'communication disorder' OR 'language disability'/exp OR 'central language imbalance' OR 'disability, language' OR 'disorder, language' OR 'language deficiency' OR 'language disorder' OR 'language disorders' OR 'language impairment' OR 'specific language disorder' OR 'specific language impairment' OR 'language disability' 'developmental language difficulties' OR 'developmental language disabilities' OR 'developmental language disability' #2. 'mass screening'/exp OR 'health screening' OR 'health screening program' OR 'health screening programme' OR 'longitudinal health screening program' OR 'longitudinal health screening programme' OR 'population screening' OR 'screening, mass' OR 'mass screening' OR 'language test'/exp OR 'language task' OR 'language tasks' OR 'language tests' OR 'test, language' OR 'language test' OR 'early diagnosis'/exp OR 'diagnosis, early' OR 'early diagnosis' OR 'screening tools' OR 'language screening test' OR 'standardized language assessment' OR 'standardized assessment' OR 'standardized test' #3.'telemedicine'/exp OR 'tele medicine' OR 'virtual medicine' OR 'telemedicine' OR 'teleconsultation'/exp OR 'long distance consultation' OR 'remote consultation' OR 'tele-consultation' OR 'telephone consultation' OR 'telephone-based consultation' OR 'teleconsultation' OR 'web-based' OR 'app-based' OR online OR 'Tele-practice' OR 'e-screening' OR 'remote screening' OR digital OR 'remote administration' #4.'preschool child'/exp OR 'child, preschool' OR 'pre-school child' OR 'pre-school going children' OR 'pre-schooler' OR 'pre-schoolers' OR 'preschool child institution' OR 'preschooler' OR 'preschool child' OR 'infant'/exp OR 'infant' OR Childhood OR 'young children' OR toddler #5. #1 AND #2 AND #3 AND #4 #5.[embase]/lim NOT ([embase]/lim AND [medline]/lim)
LILACS	#1. MH:”Transtornos do Desenvolvimento da Linguagem” OR (Language Development Disorders) OR (Trastornos del Desarrollo del Lenguaje) OR MH:C10.597.606.150.500.550$ OR MH:C23.888.592.604.150.500.550$ OR MH:”Transtornos da Comunicação” OR (Communication Disorders) OR (Trastornos de la Comunicación) OR MH:C10.597.606.150$ OR MH:C23.888.592.604.150$ OR MH:F03.625.374$ OR (developmental language difficulties) OR (developmental language disabilities) OR (developmental language disability) #2. MH:”Programas de Rastreamento” OR (Mass Screening) OR (Tamizaje Masivo) OR MH:E01.370.500$ OR MH:E05.318.308.980.438.580$ OR MH:N02.421.726.233.443$ OR MH:N05.715.360.300.800.438.500$ OR MH:N06.850.520.308.980.438.580$ OR MH:N06.850.780.500$ OR MH:SP5.312.507.332.699$ OR MH:”Testes de Linguagem” OR (Language Tests) OR (Pruebas del Lenguaje) OR MH:F04.711.513.240$ OR (screening tools) OR (language screening test) OR (standardized language assessment) OR (standardized assessment) OR (standardized test) #3. MH:”Telemedicina” OR Telemedicine OR Telemedicina OR MH:H02.403.840$ OR MH:L01.462.500.847.652$ OR MH:N04.590.374.800$ OR MH:SP2.840.065.715$ OR MH:SP2.840.566$ OR MH:”Consulta Remota” OR Remote Consultation OR MH:L01.462.500.847.652.550$ OR MH:N04.452.758.849.550$ OR MH:N04.590.374.800.550$ OR MH:SP2.840.566.030$ OR (web-based) OR (app-based) OR online OR (Tele-practice) OR (e-screening) OR (remote screening) OR digital OR (remote administration) #4. MH:”Pré-Escolar” OR (Child, Preschool) OR Preescolar OR MH:M01.060.406.448$ OR MH:”Lactente” OR Infant OR Lactante OR MH:M01.060.703$ OR Childhood OR (young children) OR toddler #5. #1 AND #2 AND #3 AND #4
PubMed/ Medline	#1.”Language Development Disorders”[Mesh] OR (Development Disorder, Language) OR (Disorder, Language Development) OR (Disorders, Language Development) OR (Language Development Disorder) OR (Developmental Disorder, Speech or Language) OR (Developmental Language Disorders) OR (Developmental Language Disorder) OR (Language Disorder, Developmental) OR (Language Disorders, Developmental) OR (Speech or Language, Developmental Disorder) OR (Language Delay) OR (Language Delays) OR “Communication Disorders”[Mesh] OR (Communication Disorder) OR (Communicative Disorders) OR (Communicative Disorder) OR (Childhood Communication Disorders) OR (Childhood Communication Disorder) OR (Communication Disorder, Childhood) OR (Communication Disorders, Childhood) OR (Communication Disabilities) OR (Communication Disability) OR (Disabilities, Communication) OR (Disability, Communication) OR (Communication Disorders, Developmental) OR (Communication Disorder, Developmental) OR (Developmental Communication Disorder) OR (Developmental Communication Disorders) OR “Language Disorders”[Mesh] OR (Language Disorder) OR (developmental language difficulties) OR (developmental language disabilities) OR (developmental language disability) #2. “Mass Screening”[Mesh] OR (Mass Screenings) OR (Screening, Mass) OR (Screenings, Mass) OR Screening OR Screenings OR (screening tools) OR “Language Tests”[Mesh] OR (Language Test) OR (Vocabulary Tests) OR (Language Comprehension Tests) OR (language screening test) OR (standardized language assessment) OR “Early Diagnosis”[Mesh] OR (Diagnosis, Early) OR (Early Detection of Disease) OR (Disease Early Detection) OR (standardized assessment) OR (standardized test) #3. “Telemedicine”[Mesh] OR (Virtual Medicine) OR (Medicine, Virtual) OR (Tele-Referral) OR (Tele Referral) OR (Tele-Referrals) OR (Mobile Health) OR (Health, Mobile) OR mHealth OR Telehealth OR eHealth OR Telecare OR (Tele-Care) OR (Tele Care) OR “Remote Consultation”[Mesh] OR (Consultation, Remote) OR Teleconsultation OR Teleconsultations OR (web-based) OR (app-based) OR online OR (Tele-practice) OR (e-screening) OR (remote screening) OR digital OR (remote administration) #4. “Child, Preschool”[Mesh] OR (Preschool Child) OR (Children, Preschool) OR (Preschool Children) OR “Infant”[Mesh] OR Infants OR Childhood OR (young children) OR toddler #5. #1 AND #2 AND #3 AND #4
Scopus	#1. (Language Development Disorders) OR (Development Disorder, Language) OR (Disorder, Language Development) OR (Disorders, Language Development) OR (Language Development Disorder) OR (Developmental Disorder, Speech or Language) OR (Developmental Language Disorders) OR (Developmental Language Disorder) OR (Language Disorder, Developmental) OR (Language Disorders, Developmental) OR (Speech or Language, Developmental Disorder) OR (Language Delay) OR (Language Delays) OR (Communication Disorders) OR (Communication Disorder) OR (Communicative Disorders) OR (Communicative Disorder) OR (Childhood Communication Disorders) OR (Childhood Communication Disorder) OR (Communication Disorder, Childhood) OR (Communication Disorders, Childhood) OR (Communication Disabilities) OR (Communication Disability) OR (Disabilities, Communication) OR (Disability, Communication) OR (Communication Disorders, Developmental) OR (Communication Disorder, Developmental) OR (Developmental Communication Disorder) OR (Developmental Communication Disorders) OR (Language Disorders) OR (Language Disorder) OR (developmental language difficulties) OR (developmental language disabilities) OR (developmental language disability) #2. (Mass Screening) OR (Mass Screenings) OR (Screening, Mass) OR (Screenings, Mass) OR Screening OR Screenings OR (screening tools) OR (Language Tests) OR (Language Test) OR (Vocabulary Tests) OR (Language Comprehension Tests) OR (language screening test) OR (standardized language assessment) OR (Early Diagnosis) OR (Diagnosis, Early) OR (Early Detection of Disease) OR (Disease Early Detection) OR (standardized assessment) OR (standardized test) #3. Telemedicine OR (Virtual Medicine) OR (Medicine, Virtual) OR (Tele-Referral) OR (Tele Referral) OR (Tele-Referrals) OR (Mobile Health) OR (Health, Mobile) OR mHealth OR Telehealth OR eHealth OR Telecare OR (Tele-Care) OR (Tele Care) OR (Remote Consultation) OR (Consultation, Remote) OR Teleconsultation OR Teleconsultations OR (web-based) OR (app-based) OR online OR (Tele-practice) OR (e-screening) OR (remote screening) OR digital OR (remote administration) #4.(Child, Preschool) OR (Preschool Child) OR (Children, Preschool) OR (Preschool Children) OR Infant OR Infants OR Childhood OR (young children) OR toddler #5. #1 AND #2 AND #3 AND #4
Web of Science	#1. (Language Development Disorders) OR (Development Disorder, Language) OR (Disorder, Language Development) OR (Disorders, Language Development) OR (Language Development Disorder) OR (Developmental Disorder, Speech or Language) OR (Developmental Language Disorders) OR (Developmental Language Disorder) OR (Language Disorder, Developmental) OR (Language Disorders, Developmental) OR (Speech or Language, Developmental Disorder) OR (Language Delay) OR (Language Delays) OR (Communication Disorders) OR (Communication Disorder) OR (Communicative Disorders) OR (Communicative Disorder) OR (Childhood Communication Disorders) OR (Childhood Communication Disorder) OR (Communication Disorder, Childhood) OR (Communication Disorders, Childhood) OR (Communication Disabilities) OR (Communication Disability) OR (Disabilities, Communication) OR (Disability, Communication) OR (Communication Disorders, Developmental) OR (Communication Disorder, Developmental) OR (Developmental Communication Disorder) OR (Developmental Communication Disorders) OR (Language Disorders) OR (Language Disorder) OR (developmental language difficulties) OR (developmental language disabilities) OR (developmental language disability) #2. (Mass Screening) OR (Mass Screenings) OR (Screening, Mass) OR (Screenings, Mass) OR Screening OR Screenings OR (screening tools) OR (Language Tests) OR (Language Test) OR (Vocabulary Tests) OR (Language Comprehension Tests) OR (language screening test) OR (standardized language assessment) OR (Early Diagnosis) OR (Diagnosis, Early) OR (Early Detection of Disease) OR (Disease Early Detection) OR (standardized assessment) OR (standardized test) #3. Telemedicine OR (Virtual Medicine) OR (Medicine, Virtual) OR (Tele-Referral) OR (Tele Referral) OR (Tele-Referrals) OR (Mobile Health) OR (Health, Mobile) OR mHealth OR Telehealth OR eHealth OR Telecare OR (Tele-Care) OR (Tele Care) OR (Remote Consultation) OR (Consultation, Remote) OR Teleconsultation OR Teleconsultations OR (web-based) OR (app-based) OR online OR (Tele-practice) OR (e-screening) OR (remote screening) OR digital OR (remote administration) #4. (Child, Preschool) OR (Preschool Child) OR (Children, Preschool) OR (Preschool Children) OR Infant OR Infants OR Childhood OR (young children) OR toddler #5. #1 AND #2 AND #3 AND #4
Google Scholar	(Language Development Disorders) OR (Language Disorders) AND (Mass Screening) AND Telemedicine AND (Preschool Child)
ProQuest	#1. (Language Development Disorders) OR (Development Disorder, Language) OR (Disorder, Language Development) OR (Disorders, Language Development) OR (Language Development Disorder) OR (Developmental Disorder, Speech or Language) OR (Developmental Language Disorders) OR (Developmental Language Disorder) OR (Language Disorder, Developmental) OR (Language Disorders, Developmental) OR (Speech or Language, Developmental Disorder) OR (Language Delay) OR (Language Delays) OR (Communication Disorders) OR (Communication Disorder) OR (Communicative Disorders) OR (Communicative Disorder) OR (Childhood Communication Disorders) OR (Childhood Communication Disorder) OR (Communication Disorder, Childhood) OR (Communication Disorders, Childhood) OR (Communication Disabilities) OR (Communication Disability) OR (Disabilities, Communication) OR (Disability, Communication) OR (Communication Disorders, Developmental) OR (Communication Disorder, Developmental) OR (Developmental Communication Disorder) OR (Developmental Communication Disorders) OR (Language Disorders) OR (Language Disorder) OR (developmental language difficulties) OR (developmental language disabilities) OR (developmental language disability) #2. (Mass Screening) OR (Mass Screenings) OR (Screening, Mass) OR (Screenings, Mass) OR Screening OR Screenings OR (screening tools) OR (Language Tests) OR (Language Test) OR (Vocabulary Tests) OR (Language Comprehension Tests) OR (language screening test) OR (standardized language assessment) OR (Early Diagnosis) OR (Diagnosis, Early) OR (Early Detection of Disease) OR (Disease Early Detection) OR (standardized assessment) OR (standardized test) #3. Telemedicine OR (Virtual Medicine) OR (Medicine, Virtual) OR (Tele-Referral) OR (Tele Referral) OR (Tele-Referrals) OR (Mobile Health) OR (Health, Mobile) OR mHealth OR Telehealth OR eHealth OR Telecare OR (Tele-Care) OR (Tele Care) OR (Remote Consultation) OR (Consultation, Remote) OR Teleconsultation OR Teleconsultations OR (web-based) OR (app-based) OR online OR (Tele-practice) OR (e-screening) OR (remote screening) OR digital OR (remote administration) #4.(Child, Preschool) OR (Preschool Child) OR (Children, Preschool) OR (Preschool Children) OR Infant OR Infants OR Childhood OR (young children) OR toddler #5. #1 AND #2 AND #3 AND #4
