# Supplementary material for: Instruments for screening and assessing oral language in preschool children via digital health: a scoping review
Source: Codas. 2026 Mar 2;37(6):e20250007. doi: 10.1590/2317-1782/e20250007en (PMC13004315; doi:10.1590/2317-1782/e20250007en)
Supplement: Translation

# Translation: Instrumentos para triagem e avaliação da linguagem oral na primeira infância via saúde digital: uma revisão de escopo

**DOI:** 10.1590/2317-1782/e20250007pt

**Authors:** Rhadimylla Nágila Pereira, Karinna Veríssimo Meira Taveira, Joseli Soares Brazorotto

**Affiliations:** 1 Programa Associado de Pós-graduação em Fonoaudiologia, Universidade Federal do Rio Grande do Norte – UFRN - Natal (RN), Brasil.; 2 Departamento de Morfologia, Universidade Federal do Rio Grande do Norte – UFRN - Natal (RN), Brasil.; 3 Departamento de Fonoaudiologia, Universidade Federal do Rio Grande do Norte – UFRN - Natal (RN), Brasil.

**Keywords:** Linguagem Infantil, Transtornos do Desenvolvimento da Linguagem, Triagem, Testes de Linguagem, Telessaúde

## Abstract

**Objetivo:**

A distribuição desigual de profissionais especializados tem motivado pesquisas em saúde digital para atender às necessidades do cuidado em linguagem oral na infância, considerando que atrasos de linguagem são queixas frequentes nessa fase. Esta revisão de escopo investigou: “Quais instrumentos são aplicáveis para triagem e avaliação da linguagem oral na primeira infância usando saúde digital?”.

**Estratégia de pesquisa:**

as bases de dados consultadas foram PubMed, Web of Science, LILACS, Embase e Scopus, além da literatura cinzenta, por meio do Google Scholar e do ProQuest Dissertations and Theses.

**Critérios de seleção:**

foram incluídos estudos que utilizaram testes de triagem e avaliação da linguagem oral por meio de teleconsulta, aplicativos e ferramentas web, sem restrição de tempo, idioma ou etnia, em contextos clínicos, educacionais e não clínicos.

**Análise dos dados:**

dos 2.435 estudos localizados, 20 compuseram a amostra final.

**Resultados:**

Onze estudos avaliaram a triagem de habilidades de linguagem e nove pesquisaram a avaliação da linguagem por meio digital. Quatorze estudos adaptaram para o formato online testes previamente validados presencialmente; quatro desenvolveram ferramentas específicas para teleconsulta e dois realizaram observações síncronas ou assíncronas sem protocolos definidos. A maioria das pesquisas foi conduzida em contextos clínicos com fonoaudiólogos, embora agentes comunitários de saúde também tenham participado de alguns estudos. A videoconferência foi a modalidade digital mais utilizada.

**Conclusão:**

os achados desta revisão reforçam a viabilidade da telessaúde para triagem e avaliação da linguagem oral, evidenciando a necessidade de mais pesquisas para consolidar a atuação baseada em evidências na telefonoaudiologia.

## INTRODUÇÃO

O emprego de triagens e avaliações da linguagem oral nos serviços de saúde tem o potencial de qualificar os encaminhamentos para a atenção especializada e, consequentemente, de reduzir as listas de espera, além de minimizar a ansiedade dos pais^(1,2)^.

Isso é particularmente relevante ao considerar os desafios impostos pelo número reduzido de profissionais de saúde e de centros especializados, bem como pela sua distribuição desigual^(3-8)^. A validação de instrumentos de triagem e avaliação da linguagem oral é essencial para diagnósticos fonoaudiológicos adequados, fomentando a ampliação do acesso aos serviços^(9-22)^.

Nesse cenário, o uso da saúde digital, particularmente as teleconsultas, tem crescido em importância, pois permite superar obstáculos geográficos de maneira flexível e conveniente para os usuários e contribui para a continuidade do cuidado e economia de recursos^(23-28)^.

Na Fonoaudiologia, há estudos nas diversas especialidades apontando para a contribuição da assistência remota na continuidade do cuidado, na autonomia do paciente e na economia de recursos, assim como indicam uma similaridade da sua eficiência em comparação ao atendimento presencial^(29-32)^.

Embora desafios como falta de infraestrutura e recursos digitais, limitação das habilidades dos profissionais e dificuldade em lidar com o atendimento de crianças tenham sido reportados^(33-35)^, o uso da telessaúde nas áreas da linguagem e fala infantil tem sido estudada nos contextos de triagem, avaliação e intervenção, com resultados favoráveis e promissores^(36-41)^. No entanto, ainda é necessário mapear os protocolos de triagem e avaliação passíveis de uso por meio da telessaúde.

Diante desse cenário e dos benefícios potenciais das ferramentas digitais na triagem e avaliação da linguagem oral em crianças, esta revisão de escopo tem como objetivo mapear os instrumentos de triagem e avaliação da linguagem oral em crianças de 0 a 6 anos, utilizando recursos de saúde digital. Dessa forma, oferece uma visão abrangente sobre instrumentos passíveis de uso para triar e avaliar a linguagem oral nesta faixa etária.

## MÉTODO

### Protocolo

A presente pesquisa se trata de uma revisão de escopo, cuja condução baseou-se na estrutura metodológica desenvolvida pelo *Joanna Briggs Institute* (*JBI*)^(42)^ e nas recomendações do *Checklist Preferred Reporting Items for Systematic Reviews* and *Meta Analysis extension for Scoping Reviews* (PRISMA-ScR)^(43)^.

A revisão segue as seis etapas propostas pelo protocolo JBI, sendo elas: formulação da questão de busca; identificação de estudos relevantes; seleção dos estudos; extração dos dados; mapeamento dos dados; agrupamento, resumo e relato/descrição dos dados encontrados.

Este estudo buscou responder à seguinte questão: Quais são os instrumentos aplicáveis para a triagem e avaliação da linguagem oral na primeira infância via saúde digital?

Para a elaboração dessa pergunta, utilizou-se o acrônimo “PCC”:

*População:* crianças de 0 a 6 anos de idade, *Conceito:* aplicação de testes e protocolos de triagem e avaliação de linguagem por meio de ferramentas de Saúde Digital; *Contexto:* ambientes clínicos, educacionais e não clínicos.

O protocolo foi registrado na plataforma https://osf.io/, com o identificador DOI 10.17605/OSF.IO/3VWJU.

### Critérios de elegibilidade

Como critérios de inclusão foram considerados estudos que utilizaram testes de triagem e de avaliação da linguagem oral na primeira infância por meio de saúde digital, a saber: teleconsulta, aplicativos, ferramentas *web*, entre outras, sem restrição de tempo, idioma ou etnia.

Foram excluídos os seguintes tipos de estudos: estudos voltados à avaliação de crianças bilíngues; revisões sistemáticas, meta-análises; correspondências, publicações de livros, diretrizes, sites, blogs, além dos títulos e resumos que não se encaixavam no escopo da revisão.

### Fontes de informação e estratégia de busca

As buscas aconteceram nas bases de dados PubMed, Web of Science, Lilacs, Embase, Scopus e na literatura cinzenta: Google Scholar e ProQuest Dissertations and Theses.

Para o levantamento dos artigos foram utilizadas as palavras-chave e operadores booleanos “Language Development Disorders” OR “language delay” AND “screening” OR “language tests” OR “standardized assessment” AND “telehealth” OR “teleconsultation” AND “child preschool” OR “toddler*”.* As estratégias completas de busca em cada base de dados, bem como na literatura cinzenta, estão detalhadas no Apêndice 1 (Apêndice 1).

### Seleção dos estudos

Fase 1: a busca foi conduzida em um único dia e, após a remoção das duplicatas no *software Mendeley*, os arquivos de cada base de dados foram anexados no *software Rayyan* (https://www.rayyan.ai/)^(44)^ para que a triagem inicial dos artigos fosse realizada.

A triagem dos artigos foi feita por dois revisores, ambos com experiência na área de linguagem oral infantil, seguindo duas fases de seleção para verificação de sua elegibilidade. Antes da seleção dos artigos, os 100 primeiros artigos da busca da base Pubmed foram inseridos no *Rayyan* para a calibração dos revisores, sendo atingido o *kappa* de 1.0, indicando concordância perfeita entre os mesmos na análise inicial.

A fase 1 foi conduzida a partir da análise dos títulos e resumos e, diante de discordâncias, uma reunião de consenso foi realizada para a tomada de decisão sobre a inclusão dos artigos conflitantes para a próxima fase.

Fase 2: nesta etapa, por sua vez, todos os artigos selecionados foram lidos na íntegra, e os dados foram extraídos utilizando um formulário previamente elaborado.

A extração dos dados foi organizada em uma tabela síntese (Tabela 1) e nos Quadros 1 e 2. Na Tabela 1 são apresentados os dados dos instrumentos: nome, habilidades avaliadas, se o protocolo corresponde a triagem ou avaliação, se foi adaptado ou específico e a referência do estudo.

**Tabela 1:** Instrumentos para triagem e avaliação da linguagem oral na primeira infância via saúde digital

Instrumento	Habilidades avaliadas	Triagem ou Avaliação	Aplicação (profissional/tempo aplicação)	Adaptado ou específico	Autores
*Parents Evaluation Developmental Status (PEDS)*	Linguagem Receptiva e Expressiva	Triagem	1)Fonoaudiólogo e ACS/NI 2) NI/ 10-15m 3)ACS/ 9 min	Adaptado como Aplicativo	1) Maleka, et al. 2016/; 2) du Toit, et al. 2021 3) Fuchs, et al. 2023
*PEDS Developmental Milestones (PEDS:DM)*	Linguagem Expressiva E Receptiva	Triagem	1)Fonoaudiólogo e ACS/NI 2)NI/ 10-15m 3)ACS/ 9 min	Adaptado como Aplicativo	1)Maleka, et al. 2016; 2) du Toit, et al.2021 3)Fuchs, et al. 2023
*Receptive-Expressive Emergent Language Test – Third Edition* (REEL-3) *Screening Kit of Language and Development* (SKOLD)	Linguagem receptiva e expressiva, comportamento de jogo/social	Triagem	Graduandos Fonoaudiologia/25 min	Adaptados para Teleconsulta Síncrona (uso de material impresso tradicional)	Ciccia, et al. 2011
*Web-based Clinical Decision Support System (CDSS)*	Recepção Sensorial, Percepção da Fala, Produção da Fala e Pragmática	Triagem	Professores + Fonoaudiólogos/ NI	Desenvolvido para Teleconsulta - Plataforma online	Ruiz, et al. 2014
Recontagem de histórias, análise, RNP, IA e questionário com os pais	Processamento fonológico, organização narrativa e competência gramatical	Triagem	Pós- graduandos em Fonoaudiologia/NI	Teleconsultas Síncrona e Assíncrona (análise de vídeos sem protocolo específico)	Guiberson, et al. 2015
Medidas de comportamento em vídeo gravadas e relatórios dos pais sobre o vocabulário	Tipologia dos gestos; jogo simbólico; MLU-W; NDW	Triagem	Triagem facilitada por fonoaudiólogo ou educador nativo de língua espanhola, com análise dos vídeos realizada por dez assistentes de pesquisa de pós-graduação/ NI	Teleconsultas Síncrona e Assíncrona (análise de vídeos sem protocolo específico)	Guiberson, et al. 2016
*PLS-4 Screening test*	Linguagem Produção de sons (articulação/fonologia/inconsistência)	Triagem	Fonoaudiólogo/ HBT - 12, 9 min SBT - 14, 1 min	Teleconsulta Síncrona	Taylor, 2017
*Telehealth Evaluation of Development for Infants (TEDI)*	Vocalizações, verbalizações e gestos	Triagem	NI	Teleconsulta Síncrona/ Assíncrona (análise de vídeos de interação)	Talbott, et al. 2020
*Language Screen*	Vocabulário Receptivo e Expressivo Repetição de Sentenças Compreensão Auditiva	Triagem	Professores e assistentes/ 10 min	Desenvolvido para Telessaúde (Aplicativo e Site)	Hulme, et al. 2024
*SRESHT Screener*	Linguagem receptiva e expressiva	Triagem	ACS e fonoaudiólogo/NI	Desenvolvido para Telessaúde (Aplicativo)	Varadharajan, et al. 2024
Peabody Picture Vocabulary Test, Revised (PPVT-R)	Vocabulário receptivo	Avaliação	Fonoaudiólogo/ NI	Teleconsulta Síncrona	Haaf, et al. 1999
Peabody Picture Vocabulary Test-3rd edition (PPVT-III) *Preschool Language Scale-4th Edition* (PLS-4)	Linguagem expressiva e receptiva	Avaliação	Fonoaudiólogo/ NI	Teleconsulta Síncrona	Eriks‐Brophy, et al. 2008
*CELF- Fourth Edition (CELF-4)*	Linguagem Receptiva e Expressiva	Avaliação	Fonoaudiólogo/ 30-60 min	Adaptado para Teleconsulta Síncrona com teste digitalizado	Waite, et al. 2010
*Peabody Picture Vocabulary Test- 4th Edition (PPVT-4)*	Vocabulário receptivo	Avaliação	NI/ 11,1 min	Adaptado para Teleconsulta Síncrona - (Plataforma online)	Anderson, 2014
*MacArthur–Bates Communicative Development Inventories (CDIs)*	Vocabulário receptivo e expressivo	Avaliação	Cuidadores/ 5,1 min	Adaptado para Teleconsulta Assíncrona (Plataforma online)	1)Makransky, et al. 2016/ 2) Kachergis, et al. 2022
*MacArthur-Bates Communicative Development Inventories* (CDIs)	Vocabulário expressivo; vocabulário receptivo	Avaliação	Cuidadores	Adaptado para Teleconsulta Assíncrona (Plataforma online)	de Mayo, et al., 2021
*Diagnostic Evaluation of Language Variation–Norm Referenced (DELV-NR) Comprehensive-Receptive and Expressive Vocabulary Test–Third Edition (CREVT-3) Test of Language Development (Primary)–Fifth Edition (TOLDP:5)*	Vocabulário Receptivo e Expressivo	Avaliação	Fonoaudiólogo/32 min (média)	Adaptados para Teleconsulta Síncrona com testes digitalizados adquiridos com as editoras	Schmitt, et al. 2022
*CELF (Clinical Evaluation of Language Fundamentals)*	Linguagem receptiva e expressiva	Avaliação	Fonoaudiólogo/ NI	Teleconsulta Síncrona	Campbell, et al. 2024

Fonte: Próprio Autor

Legenda: min = minutos; NI = não informado; MLU-W = extensão média do enunciado = NDW: número de palavras diferentes produzidas; RNP = Repetição de Não-Palavras; IA = Índice de Agramaticalidade

**Quadro 1:** Síntese descritiva dos achados dos estudos incluídos (n=20)

Autor/ ano/local	Título do artigo	Tipo de estudo	Triagem/ avaliação	Ferramenta utilizada	N amostral	Objetivo	Medidas de comparação	Resultados	Conclusões
Anderson/ 2014/ EUA	*Web-Based Telerehabilitation for Assessing Receptive Language in Children*	Estudo metodológico de validação	Avaliação	*Peabody Picture Vocabulary Test, Fourth Edition (PPVT-4)* em formato *web.*	34 crianças com idades entre 4 e 13 anos.	Avaliar a eficácia de um teste de linguagem receptiva baseado na web em comparação com o teste tradicional.	Aplicação do PPVT-4 remoto e presencial.	O teste web apresentou resultados comparáveis aos tradicionais. Não houve diferença significativa entre os dois formatos de aplicação (p > 0,05).	A telessaúde pode ser uma alternativa viável para a avaliação de linguagem em populações carentes.
Ciccia et al./ 2011/ EUA	*Improving the access of young urban children to speech, language and hearing screening via telehealth*	Estudo exploratório de viabilidade	Triagem	*Receptive-Expressive Emergent Language Test – Third Edition (REEL-3):* Este teste foi utilizado para crianças de até três anos ou com habilidades de linguagem estimadas nessa faixa etária e o *Screening Kit of Language and Development (SKOLD):* para crianças de 2,5 a 4 anos, testando a linguagem expressiva e receptiva. O teste *Preschool Language Scale 4 Articulation Screener (PLS-4)* foi aplicado nas crianças que passaram pela avaliação com o SKOLD, para verificar o inventário fonético.	411 triagens em 24 meses de estudo (263 em triagem de linguagem). No primeiro ano, 33% das crianças tinham até três anos (n=60) e no segundo ano, 40% (n=91).	Analisar a viabilidade do uso de videoconferência de baixo custo em clínicas de saúde comunitárias urbanas para triagem de fala, linguagem e audição em crianças de até seis anos de idade, além de avaliar a satisfação das famílias com a videoconferência e a confiabilidade das triagens remotas.	Foi realizada uma comparação entre as taxas de aprovação e reprovação dos testes de triagem aplicados presencialmente e por videoconferência (*Skype*).	Alta taxa de satisfação das famílias, 72% agendaram avaliações completas após falhas na triagem. Confiabilidade de 100% para triagens auditivas e de linguagem.	O uso de teleconsultas pode melhorar o acesso a serviços de triagem para famílias urbanas, superando barreiras de acesso à saúde.
Guiberson et al/ 2015/ EUA	*Accuracy of Telehealth-Administered Measures to Screen Language in Spanish-Speaking Preschoolers*	Estudo metodológico de validação	Triagem	Questionário com os pais; e filmagem de amostra de recontagem de histórias para análise da Repetição de Não Palavras (RNP) e índice de agramaticalidade (IA).	82 crianças entre 37 e 69 meses de idade (média = 53,65 meses)	Avaliar a precisão de medidas de triagem de linguagem administradas por teleconsulta a crianças de língua espanhola.	Os resultados da bateria de testes de triagem foram comparados com o teste *SPLS-4 (Spanish Preschool Language Scale*, 4ª edição)	As pontuações do NWR, índice de agramaticalidade e as medidas do SDLQ foram significativamente correlacionadas com as pontuações padrão de linguagem expressiva do SPLS-4 (r = 0,55, p < 0,01; r = −0,38, p < 0,01; vocabulário, r = 0,46, p = 0,01; questões de linguagem, r = 0,45, p = 0,01; M3L-W, r = 0,42, p = 0,01). Contudo, apenas quando as pontuações NWR foram combinadas com medidas de amostra de linguagem ou pesquisa com os pais, valores promissores de precisão de classificação que se aproximaram ou foram maiores que 0,8 foram obtidos.	Um modelo híbrido de telessaúde pode ser eficaz na triagem do desenvolvimento da linguagem em crianças de língua espanhola, especialmente quando medidas de eficiência de processamento são combinadas com questionários de pais ou amostras de linguagem.
Guiberson/ 2016/ EUA	*Telehealth Measures Screening for Developmental Language Disorders in Spanish-Speaking Toddlers*	Estudo metodológico de validação	Triagem	Medidas de comportamento em vídeo gravadas e relatórios dos pais sobre o vocabulário.	62 crianças entre 2 e 2,11 anos de idade e suas mães	Avaliar a eficácia de um modelo híbrido para triagem de desenvolvimento da linguagem em crianças hispânicas.	Os resultados da bateria de testes de triagem foram comparados com o teste *SPLS-4 (Spanish Preschool Language Scale*, 4ª edição).	As medidas de gesto e brincadeira não foram significativamente correlacionadas com as pontuações de linguagem no SPLS-4 espanhol expressivo. No entanto, ambas as medidas de amostra de linguagem (MLU-W e NDW), bem como as medidas de relato dos pais (vocabulário relatado e RM3L) foram significativamente correlacionadas (*p* ≤ 0,01) com as pontuações de linguagem. Os resultados mostraram que precisão e exatidão de classificação aprimoradas podem ser alcançadas quando o vocabulário relatado é combinado com NDW.	Modelos híbridos de triagem para a população estudada são promissores oferecendo uma alternativa viável para a triagem de linguagem.
Taylor/2017/ Austrália	*Speech and Language Screening for Children with Medical Complexity*	Dois estudos^(1)^: descritivo-exploratório (*Survey*), com fonoaudiólogos, buscando compreender barreiras para crianças com complexidade médica (CCM)* acessarem serviços fonoaudiológicos e a adoção da telessaúde na triagem de linguagem nesta população^(2)^; Estudo metodológico de validação	Triagem	Para a triagem da linguagem foram utilizados os testes- *PLS-4 Screening Test* (3 a 5 anos) ou *CELF-4 Screener* (6 a 12 anos.	(1) *Survey* aplicado em dois momentos (n=46) em 2011 e (n=47) em 2015^(2)^ 34 crianças entre 3 e 12 anos de idade	Avaliar a eficácia da triagem de fala e linguagem em crianças com complexidade médica.	Foi realizada a comparação entre os testes de triagem aplicados presencialmente e em situação remota através de dois métodos, o *Hardware-based telepractice (HBT) e System Software-based telepractice (SBT) System using consumer-grade equipment.*	A correlação foi moderada para o *PLS-4* screening test com o método *HBT* (r = 0,39) e para o *CELF-4* screener com o método *SBT* (r = 0,44), enquanto foi alta para o *PLS-4* screening test com o método *SBT* (r = 0,65) e para o *CELF-4 screener* com o método *HBT* (r = 0,68).	A triagem é uma ferramenta valiosa para melhorar o acesso a serviços de fonoaudiologia para crianças com complexidade médica.
Campbell et al./2024 /EUA	*Reliability and Feasibility of Administering a Child Language Assessment via Telehealth*	Estudo metodológico de validação	Avaliação	*CELF (Clinical Evaluation of Language Fundamentals).*A depender da idade foi aplicado o *CELF Preschool–Third Edition; CELF 5*, versão de 5 a 8 anos; CELF-5, versão de 9 a 21 anos	100 crianças de 3 a 12 anos de idade	Avaliar a eficácia da administração remota de avaliações de linguagem infantil	*CELF (Clinical Evaluation of Language Fundamentals)* no formato presencial e no remoto.	Análise de Bland-Altman, testes t pareados, Não houve diferenças significativas nas pontuações padrão entre as condições de administração.	A administração remota de avaliações de linguagem é viável e confiável, sem diferenças sistemáticas significativas.
Hulme, et al/ 2024/ Inglaterra	*LanguageScreen: The Development, Validation, and Standardization of an Automated Language Assessment App*	Estudo metodológico de validação	Triagem	*Language Screen*	350.000 crianças com idades de 3 anos e 6 meses até 8 anos e 11 meses	Desenvolver e padronizar o aplicativo móvel *“Language Screen”*, fornecendo aos professores e profissionais da educação um método rápido e confiável para avaliar a habilidade linguística das crianças	Um total de 1.156 crianças, que apresentaram as menores pontuações em suas turmas, foram reavaliadas por fonoaudiólogos usando medidas padronizadas (como a Avaliação Clínica de Fundamentos da Linguagem (*CELF*), Vocabulário Expressivo e Recordação de Sentenças e o *Renfrew Action Picture Test Information and Grammar*). As pontuações totais das avaliações de linguagem feitas pelos fonoaudiólogos correlacionaram-se fortemente com as pontuações totais do *Language Screen* (r = 0,74). Para a amostra geral foi realizada a análise psicométrica do aplicativo usando o modelo Rasch e outras ferramentas estatísticas para avaliar suas propriedades e validade.	*Language Screen* apresenta excelentes propriedades psicométricas, incluindo alta confiabilidade, bom ajuste ao modelo Rasch e mínima diferença no funcionamento dos itens entre os principais grupos de estudantes.	O *Language Screen* fornece um meio fácil de usar e confiável para identificar crianças com dificuldades de linguagem. Seu uso nas escolas pode ajudar a aumentar a conscientização dos professores sobre as variações nas habilidades linguísticas e sua importância para a prática educacional.
Kachergis, et al/ 2022/ EUA	*Online Computerized Adaptive Tests of Children’s Vocabulary Development in English and Mexican Spanish*	Estudo metodológico de validação	Avaliação	CDI-CAT	251 crianças entre 15 e 36 meses de idade	Criar versões adaptativas por computador (CAT) dos CDIs, em inglês e em espanhol.	Houve comparação entre os resultados das versões tradicionais, o *MacArthur–Bates Communicative Development Inventories* (CDIs), com a adaptativa (CDI-CAT).	O teste adaptativo mostrou ser eficaz em recuperar a habilidade dos participantes com alta correlação em relação às estimativas feitas com o teste completo. O estudo encontrou uma alta correlação (r = 0,92) entre as habilidades estimadas a partir do teste completo e do teste adaptativo.	A versão adaptativa do teste oferece uma maneira mais eficiente e ainda precisa para avaliar o vocabulário de crianças pequenas. O estudo fornece recomendações para a criação de versões adaptativas em novos idiomas e discute quando essa abordagem é apropriada.
Maleka, et al/ 2016/ África do Sul	*Developmental Screening—Evaluation of an m-Health Version of the Parents Evaluation Developmental Status Too*l	Estudo metodológico de validação	Triagem	*Parents Evaluation Developmental Status (PEDS) and PEDS: Developmental Milestones (PEDS:DM)*	207 cuidadores que estavam em uma clínica de bem-estar infantil. Os cuidadores foram divididos em uma amostra estratificada de acordo com as faixas etárias de seus filhos. As faixas etárias foram de 6 a 18 meses (69%, n = 142) e de 19 a 36 meses (31%, n = 65).	Avaliar a triagem do desenvolvimento em termos de: 1-correspondência entre o teste convencional utilizando métodos em papel realizado pelo fonoaudiólogo e o teste utilizando um aplicativo de smartphone realizado pelo agente comunitário de saúde e 2- a confiabilidade entre avaliadores entre o fonoaudiólogo e o agente comunitário de saúde.	Correspondência entre os métodos de teste (papel e aplicativo) e Confiabilidade entre avaliadores (fonoaudiólogos e ACS).	Foi encontrada uma alta correspondência positiva (100%) e negativa (96%) entre as ferramentas *PEDS* em papel e o aplicativo para smartphone, bem como entre o *SLP* e o *CHW*. Foi demonstrada quase perfeita concordância entre avaliadores (Cohen’s Kappa) entre as condições (κ = 0,873 a κ = 0,961).	Os resultados da aplicação para smartphone, operada por um Agente Comunitário de Saúde (ACS), corresponderam de perto aos padrões das ferramentas *PEDS* operadas por um profissional de saúde. ACS treinados podem realizar triagens de desenvolvimento precisas usando a versão para smartphone das ferramentas *PEDS*.
Ruiz, et al/ 2014/ Espanha	*Evaluating a Web-Based Clinical Decision Support System for Language Disorders Screening in a Nursery School*	Estudo metodológico de validação	Triagem	*Web-based Clinical Decision Support System (CDSS) - plataforma online*	146 crianças, sendo 94 de 0 a 3 anos e 52 na fase de 4 a 6 anos	O estudo avalia um sistema chamado CDSS, que é uma ferramenta digital projetada para ajudar na triagem de distúrbios de linguagem em crianças pequenas, especialmente em ambientes como escolas infantis.	Houve comparação dos resultados fornecidos pelo sistema e a avaliação de um fonoaudiólogo, contudo, não houve detalhamento sobre esta avaliação.	O processo de validação mostrou uma taxa de sucesso global de 83,6% (122/146) na avaliação da linguagem e uma taxa de 7% (7/94) de decisões do sistema não aceitas dentro da faixa etária de crianças de 0 a 3 anos. O sistema ajudou os fonoaudiólogos a identificar novas crianças com possíveis distúrbios que necessitavam de uma avaliação adicional.	Esta pesquisa demonstrou o benefício do *CDSS* baseado na *web* para monitorar o neurodesenvolvimento infantil por meio da detecção precoce de atrasos de linguagem em uma escola infantil.O sistema ajudou a identificar crianças com possíveis distúrbios de linguagem que precisavam de avaliação adicional. No entanto, o sistema precisa de ajustes, pois os terapeutas sugeriram mudanças na base de conhecimento, incluindo novas perguntas sobre produção de fala e habilidades pragmáticas.
Talbott, et al./2020/ EUA	*Brief Report: Preliminary Feasibility of the TEDI: A Novel Parent- Administered Telehealth Assessment for Autism Spectrum Disorder Symptoms in the First Year of Life*	Estudo exploratório de viabilidade	Triagem	*Telehealth Evaluation of Development for Infants (TEDI) -* durante as teleconsultas, os pais realizam atividades de brincadeira com seus bebês seguindo as instruções dos cartões de interação e os materiais fornecidos no *kit TEDI.* Essas sessões são filmadas para análise posterior. Testes utilizados:CSBS-DP Infant-Toddler Checklist (ITC); Ages and Stages Questionnaires: 3rd Edition (ASQ-3) Ages and Stages Questionnaire: Social Emotional, Second Edition (ASQ:SE-2); Autism Observation Scale for Infants (AOSI)	Crianças entre 6 e 12 meses e seus pais	Investigar a viabilidade de uma nova avaliação de telessaúde de nível 2 da comunicação social precoce e dos sintomas de TEA dos bebês, a Avaliação de Desenvolvimento de Telessaúde para Bebês (*TEDI*).	Foram realizadas comparações entre os resultados de diferentes avaliadores e entre o teste e reteste.	Os bebês tinham em média 10,23 meses na época da visita TEDI 1a (com uma faixa de 6,30 a 14,00 meses). As sessões TEDI 1a e 1b ocorreram, em média, com 22 dias de diferença (variando de 7 a 41 dias). Como esperado, houve associações significativas entre a idade dos bebês (em meses) na TEDI 1a e todas as três medidas comportamentais (pontuação total do AOSI: rs = −0,64, p = 0,034; marcadores do AOSI: rs = −0,82, p = 0,002; ECI: rs = 0,73, p = 0,02)	O estudo destaca a viabilidade do protocolo de telessaúde adaptado para triagem comportamental de bebês com sintomas de TEA, sendo a primeira a testar sistematicamente essa abordagem. Os resultados indicam que o protocolo é aceitável para os pais e pode levar a encaminhamentos mais precoces e melhores resultados para bebês com sintomas persistentes. No entanto, a falta de dados diagnósticos e o tamanho limitado da amostra são limitações significativas, e mais pesquisas com amostras maiores são necessárias para validar os resultados e avaliar a utilidade clínica do protocolo de triagem por telessaúde.
Varadharajan, et al/ 2024/ Índia	*Efficacy of a Mobile Health Screening Tool for Developmental and Speech Language Disorders*	Estudo metodológico de validação	Triagem	*SRESHT screener*, ferramenta desenvolvida pelo grupo para a triagem de desenvolvimento e da linguagem.	9 Agentes comunitários de saúde (ACS), 1 fonoaudiólogo que validou a triagem aplicada pelos ACS em 109 crianças de 0 a 6 anos.	Avaliar a eficácia do *SRESHT screener* na triagem de crianças para distúrbios de fala e linguagem.	Não houve comparação com medidas realizadas presencialmente. Apenas foi realizada a análise de confiabilidade interavaliadores.	A análise dos resultados da triagem de desenvolvimento do *RBSK* indicou uma “concordância substancial” (k = 0,62), conforme o coeficiente kappa de Cohen. Além disso, foi observada uma “concordância quase perfeita” (0,81) na comparação entre os resultados da triagem realizada pelo SLP e pelo GRW.	O uso de tecnologia pode melhorar a triagem de distúrbios de fala e linguagem em comunidades rurais.
Waite, et al./ 2010/ Austrália	*Internet-Based Telehealth Assessment of Language Using the CELF–4*	Estudo metodológico de validação	Avaliação	CELF–4 (*Clinical Evaluation of Language Fundamentals*).	25 crianças (4 com 5 anos, 8 com 6 anos, 3 com 7 anos e 10 com 8 anos) e 3 fonoaudiólogos.	Examinar a validade e confiabilidade do sistema videoconferência para a avaliação dos componentes principais de uma ferramenta de avaliação padronizada	Comparação da aplicação intra e interavaliadores da CELF-4 presencialmente e de forma remota.	Alta confiabilidade intra e interavaliador foi observada, com Coeficiente de Correlação Intraclasse variando de 0,84 a 1,00 para diferentes medidas, com 92% de concordância total entre os fonoaudiólogos participantes.	O teste CELF-4 é válido e confiável para a aplicação remota por fonoaudiólogo.
Schmitt, et al/ 2022/ EUA	*Feasibility of Assessing Expressive and Receptive Vocabulary via Telepractice for Early Elementary-Age Children With Language Impairment.*	Estudo metodológico de validação	Avaliação	*Diagnostic Evaluation of Language Variation–Norm Referenced (DELV-NR); Comprehensive-Receptive and Expressive Vocabulary Test–Third Edition (CREVT-3); Test of Language Development (Primary)–Fifth Edition (TOLDP:5).*	20 crianças com idade média de 5 anos e 11 meses	Investigar os procedimentos necessários para realizar avaliações de vocabulário receptivo e expressivo via teleprática em crianças com deficiência intelectual e avaliar a confiabilidade das pontuações dessas avaliações realizadas virtualmente.	Não houve comparação com medidas realizadas presencialmente. Apenas foi realizada a análise de confiabilidade interavaliadores.	Todos os ICCs foram fortes para cada uma das três medidas referenciadas por normas em todos os subtestes, variando de 0,937 a 0,998. Os resultados sugerem que essas adaptações resultam em forte confiabilidade entre avaliadores para pontuar as respostas dos participantes em um formato online.	Os resultados do estudo sugerem que a realização de avaliações de teleprática pode ser uma ferramenta útil e confiável para fonoaudiólogos escolares.
Fuchs, et al/ 2023/ África do Sul	*Combined screening of early childhood development, hearing and vision by community health workers using mHealth tools in a low-income-community*	Estudo observacional descritivo-analítico	Triagem	A triagem de desenvolvimento mHealth consistiu nas ferramentas Parents' Evaluation of Developmental Status (PEDS) e PEDS Developmental Milestones (PEDS: DM); triagem auditiva - hear Screen; triagem visual - aplicativo Vula Vision	63 crianças entre 4 anos e 1 mês e 6 anos e 11 meses, com a idade média de 61,78 meses (DP 6,131).	Descrever um programa de triagem combinado (desenvolvimento e sensorial – audição e visão) apoiado por mHealth conduzido por agentes de saúde comunitários com crianças de quatro a seis anos (n = 63) em centros de desenvolvimento da primeira infância em uma comunidade de baixa renda	Não houve comparação com medidas realizadas presencialmente. Realizou–se apenas a descrição das taxas de encaminhamentos.	A taxa geral de encaminhamento para a triagem de desenvolvimento foi de 30% (n = 19), para a triagem de audição foi de 6% (n = 4) e para a triagem de visão foi de 5% (n = 3). A maioria das crianças (64%; n = 40) passou em todos os três testes de triagem. Nenhuma criança foi encaminhada em ambas as triagens sensoriais. Uma criança (2%; n = 1) foi encaminhada nas triagens de desenvolvimento e visão, e duas crianças (3%; n = 2) foram encaminhadas nas triagens de desenvolvimento e audição. Algumas crianças (25%; n = 16) foram encaminhadas apenas na triagem de desenvolvimento, duas crianças (3%; n = 2) foram encaminhadas apenas na triagem de visão e duas (3%; n = 2) apenas na triagem de audição. A taxa de encaminhamento para a triagem de desenvolvimento foi significativamente maior (30%) em comparação com a taxa de encaminhamento para visão (5%; z = 4,183, p < 0,001) e audição (6%; z = 3,908, p < 0,001), respectivamente. Os questionários aplicados após a triagem, que avaliaram as perspectivas e experiências dos participantes, indicaram uma resposta positiva.	O programa integrado de triagem, apoiado por mHealth e conduzido por agentes comunitários de saúde junto com profissionais de desenvolvimento infantil, demonstrou ser viável, contribuindo para a detecção precoce de atrasos no desenvolvimento e perdas sensoriais em crianças de comunidades de baixa renda.
Makransky, et al/ 2016/ EUA	*An Item Response Theory–Based, Computerized Adaptive Testing Version of the MacArthur–Bates Communicative Development Inventory: Words & Sentences (CDI:WS)*	Estudo metodológico de validação	Avaliação	CDI-CAT	1.461 crianças com idades entre 16 e 30 meses	Investigar a viabilidade e a validade potencial de uma versão de teste adaptativo computadorizado (CAT), com o objetivo de reduzir o comprimento, mantendo a precisão da medição.	Houve comparação entre os resultados do CDI completo com a versão adaptativa (CDI-CAT).	Simulações de dados reais de CDI-CATs com pelo menos 50 itens apresentaram correlações acima de 0,95 em comparação com os resultados do CDI completo. Além disso, esses CDI-CATs mostraram correlações semelhantes com idade e nível socioeconômico em comparação ao CDI:WS completo.	Os resultados indicaram fortes evidências de que uma versão CAT do CDI:WS tem o potencial de reduzir o comprimento sem comprometer a exatidão e a precisão do instrumento completo.
deMayo, et al. /2021/EUA	*Web-CDI: A system for online administration of the MacArthurBates Communicative Development Inventories*	Estudo observacional descritivo- analítico	Avaliação	*Web-CDI*	*CDI Words & Gestures (WG) - 1620 formulários *CDI Words & Sentences (WS) - 1900 formulários	Apresentar o Web-CDI, uma ferramenta baseada na web que permite aos pesquisadores coletar dados do CDI online	Não houve comparação com medidas realizadas presencialmente. Apenas realizaram uma comparação geral entre as características demográficas obtidos em trabalhos anteriores utilizando o formulário em papel e lápis do CDI com os obtidos pelo Web-CDI	Mais de 3.500 administrações válidas dos formulários WG e WS foram coletadas no Web-CDI por mais de uma dúzia de pesquisadores nos Estados Unidos, após a aplicação de critérios de exclusão rigorosos derivados de estudos normativos anteriores. As tendências demográficas observadas em trabalhos anteriores utilizando o formulário em papel e lápis do CDI foram replicadas nos dados obtidos pelo Web-CDI.	Web-CDI é uma alternativa válida ao formulário em papel e captura resultados semelhantes
du Toit, et al/ 2021/ África do Sul	*mHealth developmental screening for preschool children in low-income communities*	Estudo metodológico de validação	Triagem	*mHealth Parents’ Evaluation of Developmental Status (PEDS) e PEDS: Developmental Milestones (PEDS: DM)*	276 participantes com crianças entre três anos e seis anos e 11 meses	Validar uma ferramenta de triagem do desenvolvimento baseada em *mHealth* para crianças entre 36 e 83 meses em comparação com um instrumento de avaliação do desenvolvimento padronizado. Também buscou determinar se existe uma associação significativa entre os critérios de encaminhamento	Foi realizada uma comparação entre os resultados da triagem com a ferramenta *mHealth PEDS* e os resultados obtidos com a Vineland Adaptive Behavior Scales, Third Edition (Vineland-3).	As ferramentas *mHealth PEDS* identificaram 237 crianças (85,9%) em risco de atraso no desenvolvimento, enquanto o *Vineland-3* identificou 221 crianças (80,1%) com atraso. A sensibilidade das ferramentas *PEDS* foi elevada (92,6%), porém a especificidade foi baixa (22,5%) ao se utilizar os critérios padronizados dos EUA. De acordo com o *PEDS: DM* (89,3%; n = 142) e o *Vineland-3* (87,1%; n = 134), as habilidades de alfabetização foram as mais atrasadas.	Apesar de as ferramentas *mHealth PEDS* apresentarem alta sensibilidade e especificidade mais baixa, elas podem ser úteis para o monitoramento do desenvolvimento infantil em crianças em idade pré-escolar em ambientes de baixa renda.
Eriks‐Brophy, et al. /2008/ Canadá	*Part of the problem or part of the solution? Communication assessments of Aboriginal children residing in remote communities using videoconferencing*	Estudo exploratório e de confiabilidade	Avaliação	*Peabody Picture Vocabulary Test-3rd edition (PPVT-III)*; *Preschool Language Scale-4th edition (PLS-4)*; subtestes do *Clinical Evaluation of Language Fundamentals4th edition (CELF-4)*; *Expressive One Word Picture Vocabulary Test (EOWPVT)*, *Goldman Fristoe Test of Articulation-2nd edition (GFTA-2)*	7 crianças entre 4;3–12;9 de idade. Essas crianças foram encaminhadas para avaliação por cuidadores ou professores devido a preocupações com relação ao desenvolvimento de sua fala e/ou linguagem	Examinar como a questão do viés nos testes pode afetar o uso de videoconferências na avaliação de fonoaudiologia para comunidades do norte e indígenas de Ontário, complementado pela coleta de percepções da comunidade sobre a utilidade dessas tecnologias.	Realizou-se uma análise de concordância percentual entre os dois fonoaudiólogos responsáveis pelo preenchimento dos testes, sendo um presente no local e outro atuando remotamente.	A concordância percentual variou de 96% a 100% para os testes de linguagem e de 66% a 100% para a medida de articulação. As crianças geralmente se ajustavam bem ao sistema, respondendo facilmente ao fonoaudiólogo remoto e seguindo as instruções do teste. No entanto, todas precisaram de um período de adaptação para entender a dinâmica do teste e como interagir com a tecnologia.	Os resultados sugerem que a videoconferência pode ser um complemento eficaz à prestação de serviços quando os procedimentos são organizados de forma a minimizar o viés na administração dos testes e na interpretação do desempenho nos testes
Haaf, et al/ 1999/Canadá	*Computer-Based Language Assessment Software: The Effects of Presentation and Response Format*	Estudo experimental	Avaliação	*Peabody Picture Vocabulary Test, Revised (PPVT-R)*	72 crianças com desenvolvimento normal entre as idades de 4:0 (anos:meses) e 8:11	Investigar os efeitos da apresentação computadorizada do PPVT, Formulário M, usando dois formatos de resposta baseados em computador.	Foi realizada comparação entre os resultados das três condições de teste (padrão, computador com trackball e com digitalização automatizada).	Os principais efeitos para a condição de teste (F = 0,217, p = 0,81) e idade (F = 0,46, p 0,50) não foram significativos, e não houve interação significativa entre esses fatores (F = 0,491, p 0,61). Esses resultados indicaram que não houve diferença significativa no desempenho do sujeito nas três condições de teste, e que o desempenho do teste em cada condição não variou significativamente em função da idade.	Os formatos de resposta adaptados da versão informatizada constituem formas estatisticamente equivalentes do PPVT-R e podem ser utilizados com as normas publicadas para este teste.

Fonte: Elaborada pela autora.

Legenda: *(CCM) = grupo de crianças com uma ou mais condições médicas crônicas e graves, limitações funcionais e necessidades de serviços/ extensivas.

O Quadro 1 apresenta as informações gerais, incluindo autores, ano, local do estudo, título do artigo, desenho do estudo, objetivo, foco na triagem ou avaliação, número amostral, idade das crianças, ferramentas utilizadas para triagem ou avaliação da linguagem, principais resultados e conclusões. Além disso, no Quadro 2, são detalhadas as características e especificidades dos protocolos adotados pelos autores, incluindo as habilidades de linguagem avaliadas, tipo de protocolo ou teste (questionário para cuidadores ou observação direta da criança), existência de validação prévia no formato convencional, formação do profissional responsável pela aplicação, disponibilidade de valores de acurácia ou validação, método de aplicação (videoconferência, plataforma online ou aplicativo), tempo de duração e contexto de aplicação (clínico ou acadêmico).

**Quadro 2:** Características específicas dos protocolos de triagem/avaliação da linguagem aplicados por meio de recursos de saúde digital (n=20)

Nome/ano/autor	Habilidades alvo	Tipo de teste	Teste já era validado no contexto presencial (S/N)	Formação do profissional	Dados de Acurácia/Validação	Método de aplicação digital	Duração da aplicação	Contexto de aplicação
Anderson/ 2014/ EUA	Vocabulário receptivo	Observação direta da criança	Sim	NI	Validade de critério - - as correlações de ordem de classificação de Spearman revelaram uma forte correlação positiva entre os dois conjuntos de pontuações brutas (r = 0,86).	O teste foi administrado através do navegador da web. O acesso do clínico e da família se deu por computador.	11,1 minutos	Clínico
Ciccia et al./ 2011/ EUA	Produção de sons (articulação); linguagem expressiva, linguagem receptiva auditiva e comportamento de jogo/social.	Questionário com os pais e observação direta da criança	Sim	Estudantes de graduação (15 semanas de aplicação) supervisionados e treinados	Não foram realizados testes de acurácia ou de validade; foi investigada apenas a confiabilidade da triagem, que foi determinada através da comparação das taxas de aprovação e reprovação para todos os componentes da triagem entre as realizadas por videoconferência e as realizadas presencialmente. No primeiro ano do estudo, a taxa de aprovação nas triagens de linguagem foi de 72% do total da amostra, enquanto no segundo ano foi de 61%.	Teleconsulta por videoconferência Quiosques de computador foram instalados nas duas clínicas, cada um equipado com *laptops* Dell com telas de 43 cm, câmeras *web* (*Microsoft Lifecam VX-3000*) e *Skype* 3.8 para *Windows*	25 minutos para a triagem completa (audição, linguagem e fala)	Clínico
Guiberson et al/ 2015/ EUA	Processamento fonológico, organização narrativa e competência gramatical	Observação direta da criança	Não	Cinco alunos de pós-graduação em fonoaudiologia	Foram estabelecidos dados de acurácia e de validade de critério. O teste de Repetição de Não Palavras (RPN) apresentou uma Área Sob a Curva de 0,83, com sensibilidade de 0,74 e especificidade de 0,75. As razões de verossimilhança positiva e negativa foram 2,94 (IC 95%: 1,73–5,0) e 0,35 (IC 95%: 0,20–0,63), respectivamente. O índice de agramaticalidade obteve valores de 0,68 para a Área Sob a Curva, 0,59 para sensibilidade, 0,67 para especificidade, 1,76 (IC 95%: 1,08–2,88) para a razão de verossimilhança positiva e 0,62 (IC 95%: 0,39–0,97) para a razão de verossimilhança negativa. O MLU-W (tradução: Extensão média do Enunciado) apresentou uma Área Sob a Curva de 0,75, sensibilidade de 0,66, especificidade de 0,91, razão de verossimilhança positiva de 7,70 (IC 95%: 2,56–23) e razão de verossimilhança negativa de 0,37 (IC 95%: 0,25–0,56). As medidas de RPN e MLU-W demonstraram boa validade concorrente. Os escores da RNP mostraram correlação significativa com os escores padrão de linguagem expressiva do SPLS-4 (r = 0,55, p < 0,01). O índice de agramaticalidade apresentou associação prática e estatisticamente significativa com os escores de linguagem expressiva do SPLS-4 (r = -0,38, p < 0,01). Foi observada uma grande magnitude de efeito para a associação da RPP e uma magnitude de efeito média foi observada para o índice de agramaticalidade, sugerindo que essas medidas podem ser úteis em sessões de triagem de linguagem via telemedicina.	Videoconferência síncrona, *videocasting* de um e-book, bem como gravação de vídeo. Para a realização dos procedimentos foram utilizados um iPad e uma câmera flip de vídeo.	NI	Clínico
Guiberson/ 2016/ EUA	Tipologia dos gestos (dêiticos, icônicos e convencionais); jogo simbólico; complexidade do jogo simbólico, MLU-W (tradução: Extensão média do Enunciado); NDW (tradução: número de palavras diferentes) produzidas	Observação direta da criança	Sim	O processo de triagem foi facilitado por um fonoaudiólogo ou educador nativo de língua espanhola *Dez assistentes de pesquisa de pós-graduação realizaram a análise dos vídeos	A acurácia foi calculada apenas para as medidas de triagem individuais mais promissoras. Para a medida NDW (tradução: número de Palavras Diferentes), a Área Sob a Curva foi de 0,82, a sensibilidade foi de 0,73, a especificidade foi de 0,83, a razão de verossimilhança positiva (±LR) foi de 4,16 (IC 95%: 2,02–8,54) e a razão de verossimilhança negativa (−LR) foi de 0,33 (IC 95%: 0,17–0,66), demonstrando valores sugestivos. Além disso, foram calculados os mesmos valores para a associação da medida NDW com as informações obtidas no questionário. Resultados: sensibilidade de 0,86 e especificidade de 0,93. A (± LR) foi de 11,51 (IC 95%: 3,83–34,63), enquanto a (-LR) foi de 0,15 (IC 95%: 0,05–0,42). Essas medidas combinadas aumentaram a precisão da triagem. Validade convergente: As medidas de gestos e brincadeiras não foram significativamente correlacionadas com as pontuações de linguagem no SPLS-4 espanhol expressivo. No entanto, ambas as medidas de amostra de linguagem, MLU-W e NDW, foram significativamente correlacionadas (*p* ≤ 0,01) com as pontuações de linguagem do teste padrão ouro.	Videoconferência assíncrona - Foram realizadas gravações de vídeo na casa da criança ou nos centros clínicos. Durante essas gravações, crianças e pais interagiam com brinquedos pré-selecionados. As gravações foram capturadas utilizando uma mini filmadora com uma taxa de amostragem de 48 kHz.	NI	Clínico
Taylor/2017/ Austrália	Linguagem; Produção de sons (articulação/fonologia/inconsistência) Avaliação Oromotora (avaliação de produção de sequências sonoras e de movimentos orais isolados e sequenciados.	Observação direta da criança	Sim	Fonoaudiólogo	Houve alta concordância entre as pontuações presenciais e as pontuações obtidas por HBT (Hardware-based telepractice) para inconsistência e tarefas oromotoras. A concordância também foi alta entre as pontuações presenciais e as pontuações obtidas por SBT (Software-based telepractice) para triagem oromotora e o PLS-4 Screening Test. No entanto, a validade foi reduzida para algumas tarefas de triagem e grupos etários, e vários sons da fala individuais não puderam ser triados de forma válida usando teleprática (todos os fricativos ou sons de alta frequência). Para as crianças em idade pré-escolar, a precisão das pontuações no Teste de Triagem PLS4 foi alta (grande correlação positiva) durante a triagem SBT e apenas média para a triagem HBT.	Vídeo Conferência síncrona*HBT (Hardware-based telepractice) system: a videoconferência foi conduzida usando os sistemas Cisco TelePresence System Quick Set C20 *SBT (Software-based telepractice) - videoconferência foi conduzida entre dois laptops (Dell Vostro 3550) com webcams externas (Logitech QuickCam Pro 9000) usando software de videoconferência gratuito (Logitech Vid HD, Cisco Jabber Video for Telepresence)	HBT: 12,9 minutos SBT: 14,1 minutos	Clínico
Campbell et al. / 2024 / EUA	Linguagem receptiva e expressiva de 3 a 5 anos foram aplicados os subtestes: Compreensão de frases, estrutura de palavras e vocabulário expressivo. Dos 5 aos 8 anos, os seguintes subtestes foram aplicados: compreensão de frases, estrutura de palavras, formulação de sentenças e recordação de frases. Dos 9 aos 12 anos, os seguintes subtestes foram aplicados: classes de palavras, formulação de frases, recontagem de sentenças e relações semânticas.	Observação direta da criança	Sim	Cinco fonoaudiólogos treinados para avaliarem a linguagem	Viés Médio: A média dos escores padrão de todas as versões do CELF teve um viés médio de 0, indicando que não houve diferença sistemática entre as condições de administração. A assimetria dos escores padrão das diferenças médias do CELF variou de -0.08 a 0.11, indicando uma distribuição normal Testes t pareados: não houve diferenças significativas nos escores padrão entre as condições, com tamanhos de efeito pequenos.A análise de Bland-Altman mostrou que a linha vermelha de igualdade (viés médio) caiu dentro do intervalo de confiança de 95%, indicando que não houve diferença significativa entre as condições.	Videoconferência:Zoom for Healthcare (Zoom Video Communications, Inc.,2023) em modelo de teleconsulta. Os fonoaudiólogos usaram os seguintes dispositivos: MacBook, Lenovo desktop, Microsoft Surface Pro, iPad, Dell laptop, enquanto as crianças utilizaram especialmente tablets e celulares. As sessões não foram gravadas.	NI	Clínico
Hulme, et al/ 2024/ Inglaterra	Vocabulário Expressivo; Vocabulário Receptivo; Repetição de Sentenças; Compreensão Auditiva	Observação direta da criança	Não	Professores e assistentes	Não foram feitas medidas de acurácia.O *Language Screen* apresentou confiabilidade boa a excelente para a escala total (ÿ = 0,92, ÿh = 0,75). O valor hierárquico ômega indica que 75% da variância da pontuação composta é atribuível à variância do fator geral do modelo bifatorial. As confiabilidades clássicas (alfas) para as subescalas separadas foram adequadas a boas (EV = 0,79, VR = 0,74, SR = 0,79, LC = 0,80). A confiabilidade teste-reteste da pontuação total do *Language Screen* foi avaliada utilizando uma amostra de crianças em turmas de acolhimento no Reino Unido (ou seja, no primeiro ano da escolaridade obrigatória). A confiabilidade teste-reteste foi boa (r = 0,78, N = 9.778).	Aplicativo e site O usuário cria uma conta, insere os dados das crianças (nome, sexo, data de nascimento) e baixa um conjunto de códigos QR. O aplicativo é instalado em um dispositivo Android ou Apple e, para iniciar a avaliação, o examinador escaneia o código QR para identificar a criança.	10 minutos	Escolar
Kachergis, et al/ 2022/ EUA	Vocabulário expressivo; vocabulário receptivo	Questionário com os pais	Sim	O questionário é realizado pelos próprios cuidadores na plataforma online. O resultado guia-os sobre a necessidade de outras avaliações.	Foram realizadas comparações entre a habilidade latente estimada (θ) das crianças a partir do CDI-CAT e a habilidade estimada via Teoria de Resposta ao Item (IRT) a partir do CDI completo. Os resultados mostram uma forte correlação entre as pontuações de habilidade estimadas pelo CDI-CAT e pelo CDI completo (r = 0,92). Também foi observada uma forte correlação entre o total de vocabulário estimado a partir do CDI completo e a habilidade estimada pelo CDI-CAT (r = 0,86). O erro médio quadrático entre as habilidades estimadas pelo CDI-CAT e o CDI completo foi de 0,55, o que sugere uma boa correspondência entre os dois métodos.O teste adaptado também apresentou alto índice de confiabilidade teste-reteste (0,95).	Plataforma online. Os cuidadores são apresentados a uma palavra por vez e devem selecionar “Sim” ou “Não” para indicar se a criança consegue falar ou compreender aquela palavra.	5,1 minutos	Clínico
Maleka, et al/ 2016/ África do Sul	Linguagem expressiva, linguagem receptiva	Questionário com os pais	Sim	Fonoaudiólogo e Agente Comunitário de Saúde	Na comparação entre métodos baseados em smartphones e papel (n = 105), a correspondência positiva alcançou 100%, a negativa 96% e a geral 100%. Entre avaliações feitas usando dois smartphones diferentes (n = 102), a correspondência positiva foi de 98%, a negativa de 98% e a geral de 100%. A correspondência negativa na faixa etária de 19 a 36 meses apresentou uma variação mais significativa, com uma taxa de 88%, sugerindo a necessidade de uma análise mais cuidadosa nessa faixa etária específica.	Aplicativo: As ferramentas PEDS foram desenvolvidas em um aplicativo para *smartphone*, usando o mesmo algoritmo das ferramentas PEDS convencionais em papel. Foram utilizados dois *smartphones Samsung Neo Trend (Android OS 4.4.1)* para instalação do aplicativo da ferramenta PEDS. Cada dispositivo foi manuseado por um fonoaudiólogo (SLP) e um agente comunitário de saúde (ACS).	NI	Clínico
Ruiz, et al/ 2014/ Espanha	Recepção Sensorial, Percepção da Fala, Produção da Fala e Pragmática.	Questionário com os professores, baseado no Denver Developmental Screening Test	Sim	Professores e Fonoaudiólogo	A Nursery School Language Therapist -NSLT (tradução: Fonoaudióloga da creche) aceitou as decisões do *Software* GADES em 93% (87/94) dos casos de 0 a 3 anos e 67% (35/52) no estágio de 4 a 6 anos. A comparação dos resultados levou a uma taxa de precisão total para o Sistema de Decisão GADES de 84% (122/146). Um total de 24 casos da amostra de 146 definiu uma taxa de não aceitação de 16% a ser reduzida com mais refinamento do sistema.	Plataforma online: *Software* GADES- fundamentado em uma ontologia que organiza as informações sobre a aquisição da linguagem da criança conforme a sua idade. O sistema inclui mais de 100 regras para gerar alertas e/ou alarmes sempre que são detectados desvios em relação ao estágio de desenvolvimento esperado para a criança.	NI	Escolar
Talbott, et al./2020/ EUA	A capacidade de comunicação e linguagem dos bebês, incluindo vocalizações, verbalizações e gestos. Desenvolvimento de habilidades sociais e comportamentais, como comunicação social.	Questionário e observação direta da criança	Sim	NI	As análises mostraram associações significativas entre a idade dos bebês e as medidas comportamentais. A pontuação total do AOSI teve uma correlação negativa com a idade dos bebês (rs = −0,64, p = .034), indicando que os bebês mais velhos apresentaram menores pontuações. O número de marcadores do AOSI também teve uma correlação negativa com a idade (rs = −0,82, p = .002). Por outro lado, a pontuação do ECI teve uma correlação positiva com a idade (rs = 0,73, p = .02), sugerindo que bebês mais velhos apresentaram melhores resultados em comunicação.	Videoconferência síncrona com análise de vídeos de interação	NI	Clínico
Varadharajan, et al/ 2024/ India	Desenvolvimento motor, cognitivo, social, linguagem., Em linguagem avalia - Linguagem Receptiva: compreensão de instruções, reconhecimento de palavras e frases e Linguagem Expressiva: Produção de palavras e frases, capacidade de usar adequadamente o vocabulário e uso de frases para expressar ideias.	Observação direta da criança	Sim (RBSK), Sim (LEST)	Agente Comunitário de Saúde e Fonoaudiólogo	Não apresentados no artigo, apenas os dados de concordância (RBSK: k = 0,62 e LEST: k = 0,68)	Aplicativo SRESHT, para dispositivos móveis (tablets, smartphones), que contém os protocolos já validados para aplicação presencial, por profissionais de saúde. No aplicativo o ACS já recebia o resultado de passa e falha, referendando uma avaliação completa.	NI	Clínico
Waite, et al./ 2010/Austrália	Foram selecionados os subtestes: conceitos e seguindo direções, estrutura de palavras, repetição de frases, formulação de frases para uma avaliação abrangente da linguagem receptiva e expressiva	Observação direta da criança	Sim	Fonoaudiólogo	Não apresentados no artigo. Foi realizado apenas a medição do índice de confiabilidade intra e interavaliadores	Videoconferência	Não foi especificado, porém, podia variar de 30 a 60 minutos	Clínico
Schmitt, et al/ 2022/ EUA	Vocabulário Receptivo e Expressivo	Observação direta da criança	Sim	Fonoaudiólogo	Não informados, mas as medidas são validadas para avaliar o vocabulário receptivo e expressivo, em inglês	Videoconferência síncrona Zoom for Healthcare (Zoom Video Communications, Inc.,2023) em modelo de teleconsulta	Média de 97 minutos (variação de 70–127 min).	Clínico
Fuchs, et al/ 2023/ África do Sul	Linguagem expressiva, linguagem receptiva	Questionário com os Profissionais de desenvolvimento da primeira infância que acompanhavam as crianças nos centros especializados	Sim	Agente Comunitário de Saúde	Não foram realizadas	Aplicativo Um smartphone Samsung A3 com o sistema operacional Android mais recente foi utilizado, com os ACSs realizando as perguntas aos profissionais de desenvolvimento da primeira infância. Após o preenchimento, o algoritmo foi automaticamente aplicado no aplicativo do smartphone, gerando os resultados, incluindo os encaminhamentos.	Triagem do desenvolvimento isolada - 9 minutos O tempo médio das triagens combinadas foi de 11,72 min (DP 3,45 min).	Clínico
Makransky, et al/ 2016/ EUA	Vocabulário expressivo; vocabulário receptivo	Questionário com os pais	Sim	O questionário é realizado pelos próprios cuidadores na plataforma *online*. O resultado guia-os sobre a necessidade de outras avaliações	O CDI-CAT com 50 itens apresentou uma correlação superior a 0,95 com o teste completo e um coeficiente de confiabilidade de 0,968. Os valores de correlação e confiabilidade aumentaram conforme aumentou-se a extensão do teste.	Plataforma online. Os cuidadores são apresentados a uma palavra por vez e devem selecionar “Sim” ou “Não” para indicar se a criança consegue falar ou compreender aquela palavra.	NI	Clínico
deMayo, et al. /2021/ EUA	Vocabulário expressivo; vocabulário receptivo	Questionário com os pais	Sim	O questionário é realizado pelos próprios cuidadores na plataforma *online*. O resultado guia-os sobre a necessidade de outras avaliações	Não foram realizadas.	Plataforma online. Os cuidadores são apresentados a uma palavra por vez e devem selecionar “Sim” ou “Não” para indicar se a criança consegue falar ou compreender aquela palavra.	NI	Clínico
du Toit, et al./ 2021/ África do Sul	Linguagem expressiva e receptiva	Questionário com os pais	Sim	NI	Os valores de sensibilidade, especificidade, valor preditivo negativo e valor preditivo positivo foram calculados para diferentes tipos de critérios de encaminhamentos.A sensibilidade das ferramentas *mHealth PEDS* diminuiu com o aumento do número de domínios de desenvolvimento no PEDS: DM, passando de 92,6% para os critérios prescritos para 74,4% para o RC2. Por outro lado, a especificidade apresentou um aumento, variando de 22,5% para 46,4%, embora tenha permanecido baixa.Ao longo dos diferentes critérios de referência (RC), o valor preditivo positivo (VPP) permaneceu alto (82,8%–84,7%), com um valor preditivo negativo (VPN) mais baixo (31,3%–40,7%). A validade convergente foi avaliada comparando os resultados do *PEDS* e do *PEDS: DM* com o *Vineland-3.* Observou-se uma associação moderadamente positiva entre o comportamento adaptativo e as habilidades de alfabetização nos testes.	Aplicativo para *Smartphones*	Entre 10 e 15 minutos	Clínico
Eriks‐Brophy, et al. /2008/ Canadá	Linguagem expressiva e receptiva	Observação direta da criança	Sim	Fonoaudiólogo	Não foram calculados dados de acurácia e de validade. Foi realizada apenas a análise de concordância percentual média entre os avaliadores.	Videoconferência síncrona	NI	Clínico
Haaf, et al/ 1999/Canadá	Vocabulário receptivo	Observação direta da criança	Sim	NI	Não foram realizadas. Apenas se calculou o grau de equivalência estatística entre os resultados a partir do teste ANOVA.	*Software*- Computador com *trackball* e com digitalização automatizada.	NI	Clínico

Fonte: Próprio Autor.

Legenda: NI= Não Informado; NR= Não Realizado..

Foi realizada consulta a três experts identificados com maior produção científica na temática durante o processo de coleta da revisão de escopo para que pudessem auxiliar na indicação de outras referências. Após a análise das indicações, um artigo foi inserido para a extração de dados. Adicionalmente, as listas de referências dos estudos previamente incluídos foram analisadas, resultando na identificação de 11 estudos com potencial para inclusão. Após a realização das fases 1 e 2 de leitura desses artigos, seis deles foram excluídos, pois abordavam exclusivamente intervenções terapêuticas ou eram voltados à população escolar.

A análise dos dados dos estudos incluídos foi realizada de forma descritiva e qualitativa.

## RESULTADOS

Para melhor organização, os resultados serão apresentados de acordo com os itens: seleção dos estudos, caracterização dos estudos, instrumentos de triagem e avaliação, desempenho dos instrumentos, habilidades avaliadas, contexto e métodos, profissionais envolvidos e recursos de saúde digital utilizados.

### Seleção dos estudos

A busca inicial identificou 2.435 estudos, dos quais 185 foram excluídos por duplicidade. Dos 2.250 restantes, 2.229 foram eliminados na triagem de títulos e resumos por inadequação aos objetivos da revisão (por exemplo: abordagem genérica do desenvolvimento, foco exclusivo em fala, ausência de dados primários ou incompatibilidade etária). Após análise integral dos 21 estudos remanescentes, sete foram excluídos, resultando em 14 artigos. Inclusões adicionais via indicação de especialistas e revisão de referências totalizaram 20 estudos. As etapas da revisão estão representadas no fluxograma mostrado na Figura 1.

**Figura 1:** Fluxograma com as etapas da revisão

### Caracterização dos estudos

Os 20 estudos incluídos compreenderam principalmente pesquisas metodológicas de validação (n=14)^(45-58)^, seguidos por estudos observacionais descritivo-analíticos^(59,60)^, exploratórios^(61-63)^ e experimentais^(64)^. Cinco artigos relataram análise de concordância interavaliadores^(48,53,55,56,62)^.

### Instrumentos de triagem e avaliação

Sete protocolos de avaliação (PPVT, CELF, CDI, PLS-4, CREVT-3, TOLDP:5, DELV-NR) e cinco de triagem (PEDS, PEDS:DM, PLS-4 screening, REEL-3, SKOLD), todos já utilizados na prática clínica presencial, foram analisados em 14 dos 20 estudos. As seis pesquisas restantes, focalizadas na triagem, se dividiram da seguinte forma: quatro delas desenvolveram ferramentas digitais para teleconsulta: *LanguageScreen* (aplicativo)^(51)^, SRESHT (aplicativo)^(48)^, *Web-based Clinical Decision Support System - CDSS* (plataforma online)^(54)^ e *Telehealth Evaluation of Development for Infants -* TEDI (protocolo observacional)^(62)^, enquanto dois estudos empregaram observação não estruturada^(46,47)^.

### Desempenho dos instrumentos

Em relação aos instrumentos já validados para aplicação presencial e que foram estudados, observou-se:

- PPVT (versões: PPVT-R, PPVT-III e PPVT-4): Resultados equivalentes à aplicação presencial, com alta concordância interavaliadores e tempo médio de 11,1 minutos^(45,63,64)^;
- CELF e CELF-4: Confiabilidade intra/interavaliadores elevada^(55)^ e resultados equivalentes aos presenciais^(50)^;
- PLS-4: Alta concordância interavaliadores^(63)^; o *Screening Test* PLS-4 correlacionou-se fortemente com métodos presenciais via *Software-Based Telepractice (SBT),* usando *laptops* (14,1 minutos de aplicação)^(49)^;
- PEDS e PEDS:DM: alta correspondência positiva (100%) e negativa (96%) entre os métodos presencial e remoto, usando aplicativo para *smartphone*, bem como concordância quase perfeita inter avaliadores (fonoaudiólogo e o Agente Comunitário de Saúde)^(53)^. No entanto, também foram observados resultados com alta sensibilidade, mas especificidade variável^(58)^;
- CDIs: Versões *online* e adaptativas (CDI-CAT) mostraram alta correlação com o teste original^(52,57)^;
- CREVT-3, TOLDP:5, DELV-NR: não houve comparação com medidas realizadas presencialmente, sendo analisadas apenas quanto à confiabilidade interavaliadores (ICC). Os ICCs apresentaram valores elevados em todos os subtestes, variando de 0,937 a 0,998, demonstrando forte concordância entre os avaliadores^(56)^;
- REEL-3, SKOLD - confiabilidade de 100%^(61)^. O estudo não apresentou detalhamentos.

Quanto às demais ferramentas, observou-se:

- *Language Screen*: apresentou excelentes propriedades psicométricas, incluindo alta confiabilidade^(51)^;
- *SRESHT:* concordância interavaliadores “quase perfeita” (0,81)^(48)^;
- *Web-based Clinical Decision Support System* (CDSS): taxa de sucesso global de 83,6%^(54)^;
- TEDI: para esse protocolo, foi desenvolvido o *kit TEDI*, contendo materiais e cartões com instruções para os pais, com o objetivo de estimular interações específicas que permitissem a pontuação das ferramentas *Autism Observation Scale for Infants* (AOSI) e *Individual Developmental Growth Indices - Early Communication Index* (ECI). Os coeficientes de correlação intraclasse indicaram uma concordância entre avaliadores de boa a excelente para as pontuações totais do ECI e do AOSI. Na confiabilidade teste-reteste, o AOSI atingiu significância estatística^(62)^. Porém, esses instrumentos ainda não são validados.

### Habilidades avaliadas

As medidas abrangeram linguagem receptiva/expressiva^(48-50,53,55,58,59,61,63)^, vocabulário^(45,51,52,56,57,60,64)^, compreensão e produção de fala^(54)^, vocalizações/gestos e jogo simbólico^(47,62)^, processamento fonológico e habilidades morfossintáticas^(46)^.

### Contexto e métodos

Em relação aos participantes, países e contexto, observou-se:

- Amostragem: Onze estudos utilizaram observação direta da criança^(45-51,55,56,63,64)^; dois combinaram questionários e observação^(61,62)^ e sete basearam-se em relatos de pais/profissionais de desenvolvimento da primeira infância ou professores^(52-54,57-60)^. O tamanho das amostras variou de 7 a 1461 participantes.
- Países: EUA (n=10)^(45-47,50,52,56,57,60-62)^, África do Sul (n=3)^(53,58,59)^, Austrália (n=2)^(49,55)^, Canadá (n=2)^(63,64)^, Espanha^(54)^, Inglaterra^(51)^ e Índia^(48)^ (n=1 cada). As publicações ocorreram entre 1999 e 2024.
- Contexto: 18 estudos em ambiente clínico^(45-50,52,53,55-64)^ e dois em ambiente escolar^(51,54)^. O tempo de aplicação variou de 5,1 a 97 minutos (apenas nove estudos reportaram o dado referente ao tempo de aplicação).

### Profissionais envolvidos

A maioria das avaliações foi conduzida por fonoaudiólogos, porém, alguns estudos relataram que os testes foram aplicados por professores^(51,54)^ e Agentes Comunitários de Saúde (ACS)^(48,53,59)^.

### Recursos de saúde digital

Teleconsultas síncronas (videoconferências) ou assíncronas predominaram (n=9)^(46,47,49,50,55,56,61-63)^, seguidas por plataformas online (n=5)^(45,52,54,57,60)^, aplicativos (n=5)^(48,51,53,58,59)^ e uso de software (n=1)^(64)^. Os dispositivos utilizados incluíram computadores, *tablets* e *smartphones.*

A síntese detalhada dos protocolos de triagem/avaliação está disponível nos Quadros 1 e 2.

Os dados dos instrumentos empregados via saúde digital para a triagem e avaliação da linguagem encontram-se na Tabela 1.

## DISCUSSÃO

A discussão apresenta três eixos principais: (i) a análise dos instrumentos de triagem e avaliação da linguagem por meio digital, considerando suas características, objetivos e processos de validação; (ii) a exploração dos recursos da saúde digital na triagem e avaliação da linguagem, com ênfase nas ferramentas tecnológicas e plataformas utilizadas; e (iii) a viabilidade e aplicabilidade dos instrumentos de triagem e avaliação da linguagem em ambiente digital, destacando desafios, benefícios e implicações para a prática clínica.

### Análise dos instrumentos de triagem e avaliação da linguagem por meio digital

Dos 20 estudos analisados, 14 consistiram em validação^(45-58)^, com foco na validade de critério – sendo oito estudos voltados para a triagem^(46-49,51,53,54,58)^ e seis para a avaliação^(45,50,52,55-57)^. Entretanto, o processo de validação mostrou-se heterogêneo. Dez estudos validaram os instrumentos comparando testes previamente validados em formato presencial com sua aplicação em contexto remoto ou confrontando os resultados com um teste considerado padrão-ouro^(45-47,49-53,57,58)^, sendo esta abordagem fundamental para a avaliação da validade de um instrumento^(9)^.

Em contraste, um estudo validou os instrumentos mediante comparação entre os resultados da triagem e a avaliação realizada por um fonoaudiólogo, sem especificar quais testes foram aplicados e quais medidas estatísticas foram empregadas^(54)^, evidenciando baixo rigor metodológico, já que o detalhamento dos testes estatísticos é essencial para que um instrumento seja considerado apto para uso clínico^(10,14)^. Adicionalmente, três estudos restringiram-se à análise da confiabilidade interavaliadores, medida complementar à validade na mensuração da qualidade de um instrumento^(48,55,56)^.

Seis estudos apresentaram caráter descritivo^(59,60)^, exploratório^(61-63)^ ou experimental^(64)^, fornecendo informações qualitativas, como o grau de satisfação das famílias^(59,61)^ e os cuidados necessários na realização das videoconferências^(63)^. Tais dados podem subsidiar a identificação de estratégias que garantam a viabilidade e eficácia das avaliações; contudo, detalhes igualmente importantes, como o tempo necessário para a aplicação, foram abordados de forma limitada^(59,62,63)^.

Observou-se variação nas habilidades linguísticas avaliadas, com predominância de pesquisas dedicadas aos protocolos de triagem^(48,49,51,53,58,59,61)^ e de avaliação^(45,50,52,55-57,60,63,64)^ da linguagem receptiva e expressiva, bem como do vocabulário, evidenciando a robustez destas medidas para identificar riscos de problemas de linguagem e avaliar de forma abrangente a linguagem oral infantil.

Para a execução dos testes, foram realizadas adaptações que incluíram a digitalização do material físico^(45,49,50,55,64)^ e a gravação do áudio das palavras e sentenças, com o objetivo de padronizar a apresentação em ambiente online^(45,55,64)^. Em alguns estudos, mesmo na ausência de gravações, buscou-se padronizar os comandos adotando-se os mesmos do formato tradicional^(50)^.

Durante os procedimentos, as crianças foram orientadas a utilizar o mouse para selecionar a imagem correspondente à palavra ouvida^(64)^, a pronunciar o número relacionado, a apontar – com a mão ou o mouse – a figura correspondente ou a desenhar na tela utilizando o recurso de anotação, permitindo que os avaliadores efetuassem a seleção^(45,49,50)^. Adicionalmente, alguns dispositivos dispunham de tela sensível ao toque, possibilitando que a criança tocasse diretamente na imagem da palavra ouvida^(55)^.

Os estudos analisados indicam que instrumentos tradicionais de avaliação e triagem da linguagem oral, tais como PPVT^(45,63,64)^, CDI MacArthur-Bates^(52,57)^, CELF^(50,55)^, PLS-4 Screening Test^(49)^, PEDS e PEDS:DM^(53,58)^, podem ser aplicados em contextos digitais, apresentando resultados comparáveis aos obtidos presencialmente, conforme confirmado por testes estatísticos. Essa constatação aponta para a possibilidade de validação de instrumentos de triagem e avaliação da linguagem oral em português brasileiro para a aplicação digital, a partir de instrumentos já validados para o contexto presencial. A escolha desses instrumentos pelos pesquisadores baseou-se no alto grau de familiaridade dos clínicos e na facilidade de adaptação do material para o formato digital^(45,64)^.

No âmbito da avaliação, o teste CELF e suas edições mais recentes avaliam a linguagem de forma mais ampla, pois envolvem linguagem receptiva e expressiva, enquanto o PPVT e os CDIs avaliam, respectivamente, o vocabulário receptivo e a combinação de vocabulário receptivo e expressivo. Contudo, por serem mais completos, tais testes demandam um tempo maior de aplicação^(55)^, o que pode representar um desafio significativo no contexto da telessaúde, em virtude das limitações de conexão^(25)^. Assim, torna-se necessária a realização de estudos que investiguem a possibilidade de aplicação em mais de uma sessão.

A qualidade da conexão constitui um ponto crítico e pode representar um desafio mesmo na aplicação de testes de triagem, como observado com o PLS-4 Screening Test^(49)^. Embora a triagem, realizada via laptops, tenha sido concluída em 14,1 minutos, distorções de imagem foram frequentes e, mesmo com resultados correlacionados aos métodos presenciais, tais problemas afetaram o grau de satisfação das famílias com o método.

No âmbito da triagem, os testes PEDS e PEDS:DM demonstraram alta correspondência entre as condições remota e presencial, mesmo que a baixa especificidade verificada^(58)^ possa implicar risco para taxas elevadas de falsos positivos. Ressalta-se, contudo, que o teste padrão-ouro utilizado, o Vineland-3, não foi validado para o contexto sul-africano, o que pode ter comprometido a precisão dos resultados quando aplicado nessa população^(12)^.

Destaca-se, pois, que a adaptação de testes amplamente consolidados no contexto presencial para o ambiente digital confere maior confiança quanto à qualidade e eficácia das ferramentas, uma vez que esses instrumentos já são conhecidos e validados em contextos tradicionais. Simultaneamente, essa familiaridade pode facilitar a adesão por parte dos fonoaudiólogos e promover o uso efetivo dos protocolos em ambientes digitais^(45,64)^.

Além dos instrumentos tradicionais adaptados, quatro estudos abordaram o desenvolvimento de ferramentas específicas para a teleconsulta^(48,51,54,62)^. Todas as ferramentas foram criadas para a triagem, incluindo duas em formato de aplicativo – *LanguageScreen* ^(51)^ e SRESHT^(48)^; uma plataforma online – o CDSS^(54)^; e um protocolo baseado na observação da interação entre cuidadores e a criança – o TEDI^(62)^. Entretanto, as evidências apresentadas permanecem limitadas, visto que apenas um estudo forneceu dados detalhados sobre a correlação com as medidas presenciais^(51)^, procedimento essencial para a validação dos instrumentos^(9)^. Os demais estudos restringiram-se a relatar índices de confiabilidade interavaliadores^(48,62)^, de teste-reteste^(62)^ ou informaram que a validação foi realizada por um fonoaudiólogo, sem o detalhamento dos procedimentos ou resultados obtidos^(54)^.

Dessa forma, a limitação dos dados referentes à correlação com medidas presenciais sugere que persistem lacunas na validação dessas ferramentas. Contudo, aspectos como o uso de ferramentas digitais de baixo custo, a possibilidade de aplicação por profissionais além do fonoaudiólogo e a inclusão de triagens realizadas no contexto escolar podem trazer contribuições relevantes, desde que futuras pesquisas aprofundem sua validação na saúde digital.

Dentre as ferramentas analisadas, apenas o PPVT e o CELF-4 encontram-se traduzidos e adaptados para o português brasileiro, embora ainda exclusivamente em formato presencial^(16,18)^. Esse cenário reforça a necessidade de ampliar pesquisas que viabilizem a tradução, adaptação e validação de instrumentos para uso tanto presenciais quanto via saúde digital^(13)^.

### Recursos da saúde digital em triagem e avaliação da linguagem

Quatro estudos utilizaram exclusivamente o celular para a triagem da linguagem oral, por meio de aplicativos^(48,53,58,59)^. Entretanto, apenas um desses estudos apresentou dados que indicavam correspondência com as avaliações presenciais^(53)^. Destaca-se que todos os aplicativos foram desenvolvidos em inglês, sendo um estudo realizado na Índia e três na África do Sul. Os protocolos utilizados nesses estudos ainda não foram traduzidos ou adaptados para o português brasileiro, evidenciando a necessidade de pesquisas que supram essa lacuna e ampliem o acesso à triagem, bem como sua aplicabilidade em diferentes contextos linguísticos e culturais.

Embora o celular constitua o principal meio de acesso à internet e possa oferecer contribuições significativas, observa-se uma disparidade notável na qualidade da conexão entre países. Em nações de baixa renda, a tecnologia 3G predomina, enquanto o acesso às redes 4G e 5G atinge apenas 52% e 4% da população, respectivamente. Em contrapartida, em países de alta renda, o acesso ao 5G chega a 84%, com maior cobertura na Europa, seguido pelas Américas. Essa disparidade também se reflete na velocidade da conexão de banda larga fixa, com dados indicando que, em países menos desenvolvidos, a velocidade é 25 vezes inferior à das economias de alta renda^(26)^.

Essas desigualdades podem comprometer tanto a acessibilidade quanto a eficácia da telessaúde, sobretudo na utilização de videoconferências síncronas, método digital mais empregado nos estudos analisados em contexto clínico. Problemas técnicos, como atrasos de áudio e vídeo, podem impactar negativamente a experiência das consultas^(24,49)^. Além das limitações decorrentes da qualidade da conexão, surgem desafios adicionais, como a necessidade de habilidades digitais dos envolvidos e de equipamentos compatíveis para assegurar o êxito do atendimento^(27,28)^. Mesmo assim, a videoconferência permanece como o método de conexão digital preferido por pacientes e clínicos, uma vez que possibilita a interação em tempo real com visualização mútua^(24)^.

No contexto escolar, os estudos analisados exploraram o uso de aplicativos e plataformas online^(51,54)^, voltados à triagem conduzida por professores. Os procedimentos foram realizados por meio de computadores^(54)^ e dispositivos móveis^(51)^, em países como Espanha e Inglaterra, respectivamente, com tempo de aplicação de 10 minutos em um dos estudos^(51)^. O acesso a ferramentas confiáveis e válidas, que promovam economia de tempo e de custos, é um dos desafios para a implementação da triagem em ambiente escolar, mesmo em países desenvolvidos^(15)^. Contribuições nessa área podem aprimorar a compreensão da viabilidade e eficácia da triagem digital, fornecendo subsídios para a elaboração de políticas públicas que integrem essa prática ao contexto educacional, facilitando a identificação precoce de dificuldades na linguagem e ampliando o acesso a intervenções especializadas realizadas por fonoaudiólogos e equipes interdisciplinares.

### Viabilidade e aplicabilidade dos instrumentos de triagem e avaliação da linguagem por meio digital

Dados relevantes para a viabilidade dos testes, como o tempo necessário para a aplicação, foram informados de forma limitada, constando em apenas nove estudos^(45,49,51,52,55,56,58,59,61)^. Esses dados, aliados às informações sobre a qualidade psicométrica, acessibilidade, custos dos instrumentos e necessidade de treinamento para sua aplicação, constituem fatores determinantes na escolha dos testes^(19)^.

No que diz respeito aos profissionais que aplicaram as triagens, dois estudos compararam os resultados obtidos pelo ACS previamente treinado e pelo fonoaudiólogo, ambos utilizando um aplicativo em smartphone para a aplicação dos testes^(48,53)^. Um desses estudos refere-se ao SRESHT^(48)^, desenvolvido especificamente para o ambiente digital, enquanto o outro consistiu em uma adaptação dos testes PEDS e PEDS:DM^(53)^. Os resultados demonstraram concordância substancial a quase perfeita entre os métodos, sugerindo que ACS treinados podem realizar triagens de desenvolvimento com precisão, o que pode representar um avanço significativo na qualificação do cuidado em saúde, com potencial de ampliação da cobertura da triagem da linguagem oral – especialmente considerando que o número de ACS é consideravelmente superior ao de fonoaudiólogos, sobretudo em países de baixa e média renda^(6-8)^.

Observa-se que a maioria das pesquisas analisadas concentrou-se na América do Norte, especialmente nos Estados Unidos, evidenciando a predominância dessa região na produção científica sobre o tema. Ainda que com objetivos distintos desta revisão de escopo, duas revisões sistemáticas também destacaram a liderança dos EUA em pesquisas sobre telessaúde na fonoaudiologia^(40,41)^, reforçando a necessidade de ampliar investigações dessa natureza em países subdesenvolvidos e em desenvolvimento, onde a implementação de soluções digitais pode impactar significativamente a ampliação do acesso aos serviços de saúde.

Por outro lado, não foram identificados estudos oriundos da América Latina, Oriente Médio ou Ásia Oriental. Para a aplicação desses protocolos na realidade brasileira, é imprescindível a validação dos instrumentos para o português brasileiro, considerando a população-alvo e as especificidades regionais, bem como os contextos sociais, linguísticos e educacionais^(20)^. Ademais, o acesso à internet de qualidade deve ser considerado.

Portanto, futuras pesquisas devem priorizar a apresentação detalhada dessas variáveis, permitindo uma análise aprofundada da viabilidade e aplicabilidade dos instrumentos de triagem e avaliação da linguagem oral infantil no contexto da saúde digital, especialmente considerando o acesso de populações vulneráveis e de regiões com recursos limitados.

## CONCLUSÃO

Foram identificados onze estudos que investigaram instrumentos de triagem e nove de avaliação da linguagem oral na primeira infância.

A equiparação entre os resultados das avaliações por meio da telessaúde e os métodos convencionais presenciais sugere que essas tecnologias podem ser integradas de forma eficaz nas práticas fonoaudiológicas e, no caso da triagem, por outros profissionais de saúde, desde que haja o devido treinamento e o seguimento das boas práticas em telefonoaudiologia.

Mesmo que os instrumentos digitais para triagem e avaliação da linguagem oral infantil tenham apresentado confiabilidade e aplicabilidade em suas realidades, ainda há a necessidade de validação, considerando os contextos linguísticos e culturais dos países.

A análise deste estudo revela que a expansão da saúde digital para a triagem e avaliação da linguagem oral no Brasil requer, além de avanços científicos, melhorias abrangentes. Isso inclui a implementação de políticas públicas que garantam o acesso à saúde digital, a disponibilização de internet de qualidade e a formação adequada de profissionais de saúde e fonoaudiólogos para proporcionar cuidados com o uso das tecnologias.

## Apêndice 1 Estratégias de busca na literatura

**Table:**

Database	Search (August 9th 2024)
Embase	#1. 'developmental language disorder'/exp OR 'language development disorder' OR 'language development disorders' OR 'developmental language disorder' OR 'communication disorder'/exp OR 'communication disease' OR 'communication disorders' OR 'communication problem' OR 'communicative disorder' OR 'social communication disorder' OR 'communication disorder' OR 'language disability'/exp OR 'central language imbalance' OR 'disability, language' OR 'disorder, language' OR 'language deficiency' OR 'language disorder' OR 'language disorders' OR 'language impairment' OR 'specific language disorder' OR 'specific language impairment' OR 'language disability' 'developmental language difficulties' OR 'developmental language disabilities' OR 'developmental language disability' #2. 'mass screening'/exp OR 'health screening' OR 'health screening program' OR 'health screening programme' OR 'longitudinal health screening program' OR 'longitudinal health screening programme' OR 'population screening' OR 'screening, mass' OR 'mass screening' OR 'language test'/exp OR 'language task' OR 'language tasks' OR 'language tests' OR 'test, language' OR 'language test' OR 'early diagnosis'/exp OR 'diagnosis, early' OR 'early diagnosis' OR 'screening tools' OR 'language screening test' OR 'standardized language assessment' OR 'standardized assessment' OR 'standardized test' #3.'telemedicine'/exp OR 'tele medicine' OR 'virtual medicine' OR 'telemedicine' OR 'teleconsultation'/exp OR 'long distance consultation' OR 'remote consultation' OR 'tele-consultation' OR 'telephone consultation' OR 'telephone-based consultation' OR 'teleconsultation' OR 'web-based' OR 'app-based' OR online OR 'Tele-practice' OR 'e-screening' OR 'remote screening' OR digital OR 'remote administration' #4.'preschool child'/exp OR 'child, preschool' OR 'pre-school child' OR 'pre-school going children' OR 'pre-schooler' OR 'pre-schoolers' OR 'preschool child institution' OR 'preschooler' OR 'preschool child' OR 'infant'/exp OR 'infant' OR Childhood OR 'young children' OR toddler #5. #1 AND #2 AND #3 AND #4 #5.[embase]/lim NOT ([embase]/lim AND [medline]/lim)
LILACS	#1. MH:”Transtornos do Desenvolvimento da Linguagem” OR (Language Development Disorders) OR (Trastornos del Desarrollo del Lenguaje) OR MH:C10.597.606.150.500.550$ OR MH:C23.888.592.604.150.500.550$ OR MH:”Transtornos da Comunicação” OR (Communication Disorders) OR (Trastornos de la Comunicación) OR MH:C10.597.606.150$ OR MH:C23.888.592.604.150$ OR MH:F03.625.374$ OR (developmental language difficulties) OR (developmental language disabilities) OR (developmental language disability) #2. MH:”Programas de Rastreamento” OR (Mass Screening) OR (Tamizaje Masivo) OR MH:E01.370.500$ OR MH:E05.318.308.980.438.580$ OR MH:N02.421.726.233.443$ OR MH:N05.715.360.300.800.438.500$ OR MH:N06.850.520.308.980.438.580$ OR MH:N06.850.780.500$ OR MH:SP5.312.507.332.699$ OR MH:”Testes de Linguagem” OR (Language Tests) OR (Pruebas del Lenguaje) OR MH:F04.711.513.240$ OR (screening tools) OR (language screening test) OR (standardized language assessment) OR (standardized assessment) OR (standardized test) #3. MH:”Telemedicina” OR Telemedicine OR Telemedicina OR MH:H02.403.840$ OR MH:L01.462.500.847.652$ OR MH:N04.590.374.800$ OR MH:SP2.840.065.715$ OR MH:SP2.840.566$ OR MH:”Consulta Remota” OR Remote Consultation OR MH:L01.462.500.847.652.550$ OR MH:N04.452.758.849.550$ OR MH:N04.590.374.800.550$ OR MH:SP2.840.566.030$ OR (web-based) OR (app-based) OR online OR (Tele-practice) OR (e-screening) OR (remote screening) OR digital OR (remote administration) #4. MH:”Pré-Escolar” OR (Child, Preschool) OR Preescolar OR MH:M01.060.406.448$ OR MH:”Lactente” OR Infant OR Lactante OR MH:M01.060.703$ OR Childhood OR (young children) OR toddler #5. #1 AND #2 AND #3 AND #4
PubMed/ Medline	#1.”Language Development Disorders”[Mesh] OR (Development Disorder, Language) OR (Disorder, Language Development) OR (Disorders, Language Development) OR (Language Development Disorder) OR (Developmental Disorder, Speech or Language) OR (Developmental Language Disorders) OR (Developmental Language Disorder) OR (Language Disorder, Developmental) OR (Language Disorders, Developmental) OR (Speech or Language, Developmental Disorder) OR (Language Delay) OR (Language Delays) OR “Communication Disorders”[Mesh] OR (Communication Disorder) OR (Communicative Disorders) OR (Communicative Disorder) OR (Childhood Communication Disorders) OR (Childhood Communication Disorder) OR (Communication Disorder, Childhood) OR (Communication Disorders, Childhood) OR (Communication Disabilities) OR (Communication Disability) OR (Disabilities, Communication) OR (Disability, Communication) OR (Communication Disorders, Developmental) OR (Communication Disorder, Developmental) OR (Developmental Communication Disorder) OR (Developmental Communication Disorders) OR “Language Disorders”[Mesh] OR (Language Disorder) OR (developmental language difficulties) OR (developmental language disabilities) OR (developmental language disability) #2. “Mass Screening”[Mesh] OR (Mass Screenings) OR (Screening, Mass) OR (Screenings, Mass) OR Screening OR Screenings OR (screening tools) OR “Language Tests”[Mesh] OR (Language Test) OR (Vocabulary Tests) OR (Language Comprehension Tests) OR (language screening test) OR (standardized language assessment) OR “Early Diagnosis”[Mesh] OR (Diagnosis, Early) OR (Early Detection of Disease) OR (Disease Early Detection) OR (standardized assessment) OR (standardized test) #3. “Telemedicine”[Mesh] OR (Virtual Medicine) OR (Medicine, Virtual) OR (Tele-Referral) OR (Tele Referral) OR (Tele-Referrals) OR (Mobile Health) OR (Health, Mobile) OR mHealth OR Telehealth OR eHealth OR Telecare OR (Tele-Care) OR (Tele Care) OR “Remote Consultation”[Mesh] OR (Consultation, Remote) OR Teleconsultation OR Teleconsultations OR (web-based) OR (app-based) OR online OR (Tele-practice) OR (e-screening) OR (remote screening) OR digital OR (remote administration) #4. “Child, Preschool”[Mesh] OR (Preschool Child) OR (Children, Preschool) OR (Preschool Children) OR “Infant”[Mesh] OR Infants OR Childhood OR (young children) OR toddler #5. #1 AND #2 AND #3 AND #4
Scopus	#1. (Language Development Disorders) OR (Development Disorder, Language) OR (Disorder, Language Development) OR (Disorders, Language Development) OR (Language Development Disorder) OR (Developmental Disorder, Speech or Language) OR (Developmental Language Disorders) OR (Developmental Language Disorder) OR (Language Disorder, Developmental) OR (Language Disorders, Developmental) OR (Speech or Language, Developmental Disorder) OR (Language Delay) OR (Language Delays) OR (Communication Disorders) OR (Communication Disorder) OR (Communicative Disorders) OR (Communicative Disorder) OR (Childhood Communication Disorders) OR (Childhood Communication Disorder) OR (Communication Disorder, Childhood) OR (Communication Disorders, Childhood) OR (Communication Disabilities) OR (Communication Disability) OR (Disabilities, Communication) OR (Disability, Communication) OR (Communication Disorders, Developmental) OR (Communication Disorder, Developmental) OR (Developmental Communication Disorder) OR (Developmental Communication Disorders) OR (Language Disorders) OR (Language Disorder) OR (developmental language difficulties) OR (developmental language disabilities) OR (developmental language disability) #2. (Mass Screening) OR (Mass Screenings) OR (Screening, Mass) OR (Screenings, Mass) OR Screening OR Screenings OR (screening tools) OR (Language Tests) OR (Language Test) OR (Vocabulary Tests) OR (Language Comprehension Tests) OR (language screening test) OR (standardized language assessment) OR (Early Diagnosis) OR (Diagnosis, Early) OR (Early Detection of Disease) OR (Disease Early Detection) OR (standardized assessment) OR (standardized test) #3. Telemedicine OR (Virtual Medicine) OR (Medicine, Virtual) OR (Tele-Referral) OR (Tele Referral) OR (Tele-Referrals) OR (Mobile Health) OR (Health, Mobile) OR mHealth OR Telehealth OR eHealth OR Telecare OR (Tele-Care) OR (Tele Care) OR (Remote Consultation) OR (Consultation, Remote) OR Teleconsultation OR Teleconsultations OR (web-based) OR (app-based) OR online OR (Tele-practice) OR (e-screening) OR (remote screening) OR digital OR (remote administration) #4.(Child, Preschool) OR (Preschool Child) OR (Children, Preschool) OR (Preschool Children) OR Infant OR Infants OR Childhood OR (young children) OR toddler #5. #1 AND #2 AND #3 AND #4
Web of Science	#1. (Language Development Disorders) OR (Development Disorder, Language) OR (Disorder, Language Development) OR (Disorders, Language Development) OR (Language Development Disorder) OR (Developmental Disorder, Speech or Language) OR (Developmental Language Disorders) OR (Developmental Language Disorder) OR (Language Disorder, Developmental) OR (Language Disorders, Developmental) OR (Speech or Language, Developmental Disorder) OR (Language Delay) OR (Language Delays) OR (Communication Disorders) OR (Communication Disorder) OR (Communicative Disorders) OR (Communicative Disorder) OR (Childhood Communication Disorders) OR (Childhood Communication Disorder) OR (Communication Disorder, Childhood) OR (Communication Disorders, Childhood) OR (Communication Disabilities) OR (Communication Disability) OR (Disabilities, Communication) OR (Disability, Communication) OR (Communication Disorders, Developmental) OR (Communication Disorder, Developmental) OR (Developmental Communication Disorder) OR (Developmental Communication Disorders) OR (Language Disorders) OR (Language Disorder) OR (developmental language difficulties) OR (developmental language disabilities) OR (developmental language disability) #2. (Mass Screening) OR (Mass Screenings) OR (Screening, Mass) OR (Screenings, Mass) OR Screening OR Screenings OR (screening tools) OR (Language Tests) OR (Language Test) OR (Vocabulary Tests) OR (Language Comprehension Tests) OR (language screening test) OR (standardized language assessment) OR (Early Diagnosis) OR (Diagnosis, Early) OR (Early Detection of Disease) OR (Disease Early Detection) OR (standardized assessment) OR (standardized test) #3. Telemedicine OR (Virtual Medicine) OR (Medicine, Virtual) OR (Tele-Referral) OR (Tele Referral) OR (Tele-Referrals) OR (Mobile Health) OR (Health, Mobile) OR mHealth OR Telehealth OR eHealth OR Telecare OR (Tele-Care) OR (Tele Care) OR (Remote Consultation) OR (Consultation, Remote) OR Teleconsultation OR Teleconsultations OR (web-based) OR (app-based) OR online OR (Tele-practice) OR (e-screening) OR (remote screening) OR digital OR (remote administration) #4.(Child, Preschool) OR (Preschool Child) OR (Children, Preschool) OR (Preschool Children) OR Infant OR Infants OR Childhood OR (young children) OR toddler #5. #1 AND #2 AND #3 AND #4
Google Scholar	(Language Development Disorders) OR (Language Disorders) AND (Mass Screening) AND Telemedicine AND (Preschool Child)
ProQuest	#1. (Language Development Disorders) OR (Development Disorder, Language) OR (Disorder, Language Development) OR (Disorders, Language Development) OR (Language Development Disorder) OR (Developmental Disorder, Speech or Language) OR (Developmental Language Disorders) OR (Developmental Language Disorder) OR (Language Disorder, Developmental) OR (Language Disorders, Developmental) OR (Speech or Language, Developmental Disorder) OR (Language Delay) OR (Language Delays) OR (Communication Disorders) OR (Communication Disorder) OR (Communicative Disorders) OR (Communicative Disorder) OR (Childhood Communication Disorders) OR (Childhood Communication Disorder) OR (Communication Disorder, Childhood) OR (Communication Disorders, Childhood) OR (Communication Disabilities) OR (Communication Disability) OR (Disabilities, Communication) OR (Disability, Communication) OR (Communication Disorders, Developmental) OR (Communication Disorder, Developmental) OR (Developmental Communication Disorder) OR (Developmental Communication Disorders) OR (Language Disorders) OR (Language Disorder) OR (developmental language difficulties) OR (developmental language disabilities) OR (developmental language disability) #2. (Mass Screening) OR (Mass Screenings) OR (Screening, Mass) OR (Screenings, Mass) OR Screening OR Screenings OR (screening tools) OR (Language Tests) OR (Language Test) OR (Vocabulary Tests) OR (Language Comprehension Tests) OR (language screening test) OR (standardized language assessment) OR (Early Diagnosis) OR (Diagnosis, Early) OR (Early Detection of Disease) OR (Disease Early Detection) OR (standardized assessment) OR (standardized test) #3. Telemedicine OR (Virtual Medicine) OR (Medicine, Virtual) OR (Tele-Referral) OR (Tele Referral) OR (Tele-Referrals) OR (Mobile Health) OR (Health, Mobile) OR mHealth OR Telehealth OR eHealth OR Telecare OR (Tele-Care) OR (Tele Care) OR (Remote Consultation) OR (Consultation, Remote) OR Teleconsultation OR Teleconsultations OR (web-based) OR (app-based) OR online OR (Tele-practice) OR (e-screening) OR (remote screening) OR digital OR (remote administration) #4.(Child, Preschool) OR (Preschool Child) OR (Children, Preschool) OR (Preschool Children) OR Infant OR Infants OR Childhood OR (young children) OR toddler #5. #1 AND #2 AND #3 AND #4
